# Extensive regulation of the non-coding transcriptome by hypoxia: role of HIF in releasing paused RNApol2

**DOI:** 10.1002/embr.201337642

**Published:** 2013-12-22

**Authors:** Hani Choudhry, Johannes Schödel, Spyros Oikonomopoulos, Carme Camps, Steffen Grampp, Adrian L Harris, Peter J Ratcliffe, Jiannis Ragoussis, David R Mole

**Affiliations:** 1 The Wellcome Trust Centre for Human Genetics, University of OxfordOxford, UK; 2 Department of Biochemistry, Faculty of Science, King Abdulaziz UniversityJeddah, Saudi Arabia; 3 The Henry Wellcome Building for Molecular Physiology, University of OxfordOxford, UK; 4 Department of Nephrology and Hypertension, Friedrich-Alexander-University Erlangen- NurembergErlangen, Germany; 5 The Weatherall Institute of Molecular Medicine, University of OxfordOxford, UK; 6 McGill University and Genome Quebec Innovation CentreMontreal, Canada; 7 BSRC Alexander FlemingAthens, Greece

**Keywords:** HIF, hypoxia, non-coding, RNApol2, transcription

## Abstract

Hypoxia is central to both ischaemic and neoplastic diseases. However, the non-coding transcriptional response to hypoxia is largely uncharacterized. We undertook integrated genomic analyses of both non-coding and coding transcripts using massively parallel sequencing and interfaced this data with pan-genomic analyses of hypoxia-inducible factor (HIF) and RNApol2 binding in hypoxic cells. These analyses revealed that all classes of RNA are profoundly regulated by hypoxia and implicated HIF as a major direct regulator of both the non-coding and coding transcriptome, acting predominantly through release of pre-bound promoter-paused RNApol2. These findings indicate that the transcriptional response to hypoxia is substantially more extensive than previously considered.

## Introduction

Cells respond to physiological insults by altering their transcriptional output. Hypoxia (low oxygen level) is an important environmental stress with a central role in many physiological responses including adaptation to altitude, exercise, growth and development, as well as major pathophysiological processes such as ischaemic vascular disease, inflammation, wound healing and cancer 1. The transcription factor, hypoxia-inducible factor (HIF) orchestrates many of these responses to limit the disturbance of oxygen homeostasis or promote repair processes, through the transactivation of protein-coding genes with key roles in pathways such as apoptosis, differentiation, proliferation, energy metabolism and growth factor production 2. Given the fundamental role of disturbed oxygen homeostasis in human disease and the potential for therapeutic manipulation of the HIF pathway, much interest has focused on understanding the extent and architecture of these pathways.

To date, pan-genomic analyses of the transcriptional response to hypoxia using microarrays have focussed on protein-coding RNAs (mRNAs) and some microRNAs (miRNAs) 3 4. However, recent genomic analyses have transformed our perspective of regulatory transcriptional networks. It is now recognised that mRNA forms only part of a much broader transcriptional output that includes even greater numbers of RNAs that are not translated to protein 5. These ‘non-coding’ RNAs have important regulatory roles that further shape the transcriptional output of the cell. They include short non-coding RNA (<200 nucleotides) such as micro (miRNA), small nuclear/nucleolar (snRNA), piwi-interacting RNAs (piwiRNAs), and transfer RNAs (tRNAs) as well as long non-coding RNAs (lncRNA) (>200 nucleotides). Recent work has demonstrated altered non-coding RNA expression in many types of cancer and revealed functions in cell cycle regulation, apoptosis, carcinogenesis and metastasis 5 6. However, little is known about the mechanisms regulating the expression of the non-coding transcriptome.

Here, we provide the first pan-genomic analysis of both non-coding and coding transcriptional responses to hypoxia, including lncRNAs, miRNAs, piwiRNAs, snRNAs, and tRNAs. The analysis reveals marked bidirectional hypoxia-inducible changes in transcript abundance across all classes of RNA and demonstrates the importance of HIF in regulating non-coding as well as coding transcriptional responses to hypoxia. Correlation with changes in histone marks and RNA polymerase 2 (RNApol2) positioning, indicates that activation of gene expression by HIF occurs commonly through long-range interactions that cause the release of pre-bound, promoter-paused RNApol2.

## Results and Discussion

### Extensive regulation of the coding and non-coding transcriptome by hypoxia

**Figure 1:**
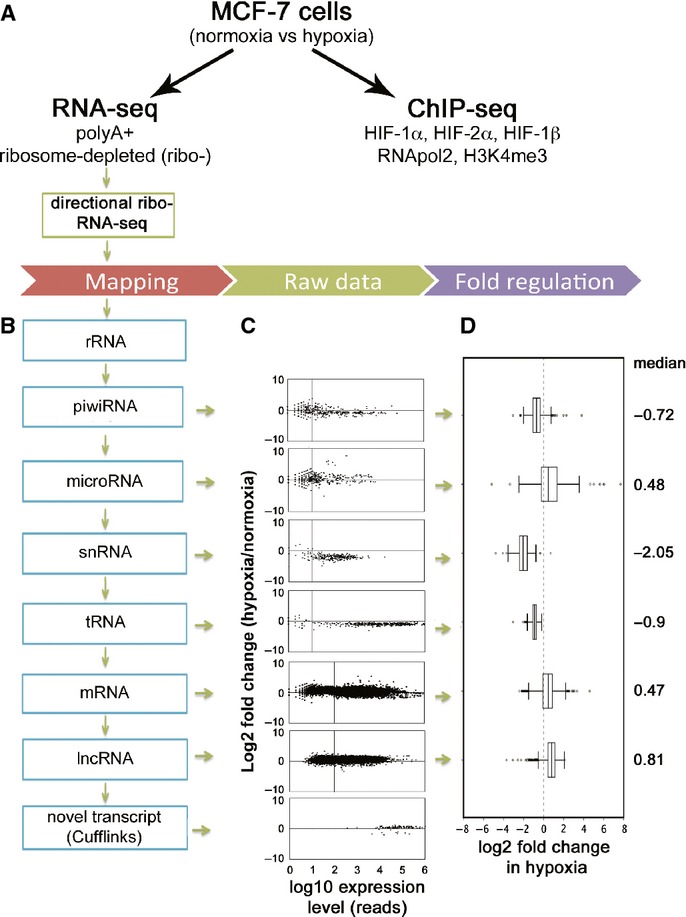
Extensive regulation of transcript abundance by hypoxia RNA-seq was performed following 24 h culture in 21% (normoxia) or 1% (hypoxia) ambient oxygen.Outline of the pipeline for mapping ribosomal depleted directional RNA-seq reads.Raw data plotted as log2 fold-change by hypoxia on the vertical axis versus expression level on the horizontal axis. Vertical lines denote thresholds (higher for classes of longer RNAs) to select transcripts for further analysis.Box-and-whisker plots of log2 fold-change by hypoxia for filtered transcripts in each class of RNA. The vertical dotted line denotes no fold-change.

To gain a global overview of the transcriptional response to hypoxia, we integrated expression profile and chromatin maps of human breast cancer MCF-7 cells incubated in either 21% ambient oxygen (normoxia) or 1% ambient oxygen (hypoxia) (Fig1A). Independent directional and nondirectional cDNA libraries were generated from polyadenylated RNA (polyA+) and total RNA depleted of ribosomal RNAs (ribo-) and analysed by high-throughput sequencing. For transcripts present in both data sets, expression levels determined by the two methods showed a high degree of correlation (supplementary Fig S1), although a subset of transcripts showed incomplete adenylation 7.

To determine transcript abundance across all classes of RNA, the ribosome depleted, directional RNA-seq dataset was mapped sequentially to databases of different RNA classes (Fig1B). 42989 non-ribosomal transcripts were detected in normoxia and 43169 in hypoxia. The raw data for each class is presented in Fig1C, whilst Fig1D shows the median fold-change in hypoxia, together with the interquartile and full ranges, after exclusion of low-abundance transcripts where this ratio might be unreliable. This demonstrates that all classes of RNA are regulated by hypoxia, but reveals major class-specific differences. Several classes of transcript (snRNAs, tRNAs and piwiRNAs) demonstrated overall downregulation, whereas mRNAs, lncRNAs and miRNAs demonstrated overall upregulation. To confirm these findings, representative samples of snRNA, mRNA and lncRNA transcripts exhibiting regulation close to the median values were tested by quantitative (q)PCR. This analysis recapitulated differences between the classes, consistent with the finding of overall class-specific regulation (supplementary Fig S2). In addition to these global changes in hypoxia, a number of transcripts in each category show strong up- or downregulation, ranging from 15-fold up to 8-fold down (piwiRNA), >100-fold up to 30-fold down (miRNA) and 4-fold up to 12-fold down (lncRNA) (supplementary Table S1).

Several microarray-based pan-genomic analyses have described heterogeneous responses in the miRNA response to hypoxia depending on cell type, hypoxic stimulus and profiling platform – reviewed in McCormick *et al* 4. A number of these miRNAs(including mir-210) were also increased in our study (supplementary Table S2). In addition, upregulated miRNAs from our analysis were also induced across a panel of breast cancer cell lines (supplementary Fig S3).

As few lncRNAs have been included in previous pan-genomic analyses our data identifies lncRNAs as a new class of transcript that are commonly regulated by hypoxia. The majority of these transcripts remain functionally uncharacterized. However, two lncRNAs (NEAT1 and MALAT1) included in a previous microarray-based analysis 8 were the most strongly upregulated lncRNAs in our dataset and were also upregulated in a panel of breast cancer cell lines (supplementary Fig S4). NEAT1 is a component of interchromatin-paraspeckles, which regulate nuclear retention of Adenosine to Inosine (A-to-I) edited RNAs 9. MALAT1 promotes cellular proliferation, is associated with tumour growth, metastasis and recurrence and is frequently upregulated or mutated in solid tumours 10. In addition, H19, another hypoxia-inducible oncogenic lncRNA 11 was also induced in our analysis but did not have a HIF-binding site close by.

### Hypoxic induction of non-annotated intergenic and antisense transcripts

A proportion of reads did not map to an annotated transcript of any class. To define whether these represent previously non-annotated transcripts, they were first examined for contiguity, using the Cufflinks de novo transcript assembler. This identified 91 novel RNA transcripts with a median length of 9988 bp (range 269–38 2549 bp) many of which were upregulated in hypoxia (supplementary Tables S3 and S4) and were confirmed by qPCR (supplementary Fig S5).

In keeping with assignment as *bona fide* non-annotated transcripts, reference to UCSC data (http://genome.ucsc.edu/cgi-bin/hgTrackUi?hgsid=336849981&g=cpgIslandExt) revealed that 50 (55%) have a CpG Island within 1000 bp of the putative promoter. All 91 transcripts were also detected in the independently derived polyA+ RNA-seq dataset. Of the 91 non-annotated transcripts, 37 did not overlap with any annotated RefSeq gene, and were therefore classified as non-annotated intergenic transcripts (supplementary Table S3). All had a low coding potential 12, indicating that they belong to the lncRNA class of RNA. The remaining 54 overlapped with a previously documented RNA, but were expressed from the opposite DNA strand indicating that they are non-annotated anti-sense transcripts (NATS) (supplementary Table S4). Anti-sense transcripts may act in *cis* to regulate expression of the overlapping sense transcript 13. Interestingly, the majority were upregulated by hypoxia, induction being commonly associated either with counter-regulation (e.g. HIF-1α, TBX2) or with co-regulation of the overlapping sense transcript (e.g. SPAG4, CELSR2) (supplementary Fig S6). Thus, hypoxia-induced antisense transcripts are allied to both induction and repression in *cis* adding further tiers to regulation of the transcriptome by hypoxia.

### Transcriptional regulation of long non-coding RNAs by HIF

**Figure 2:**
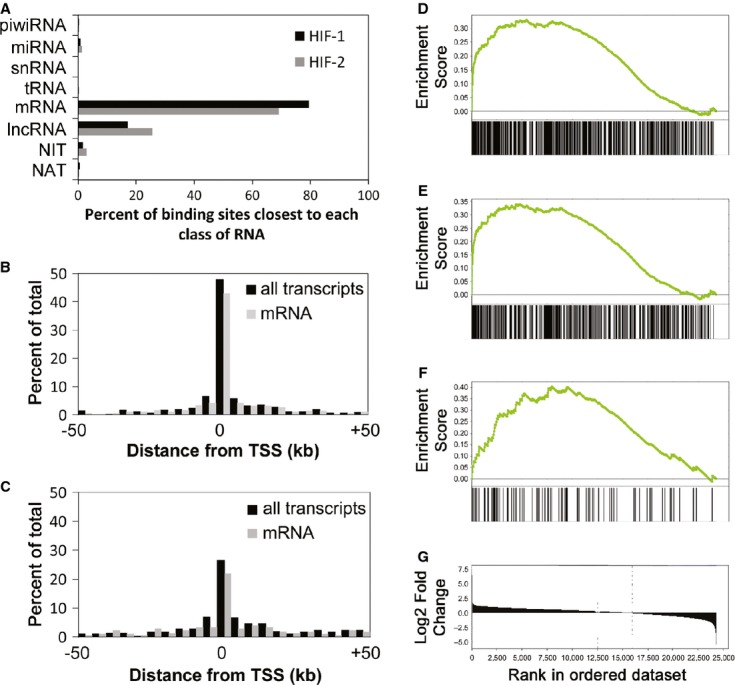
HIF binding upregulates both the coding and non-coding transcriptome A–C she proportion of HIF-1α and HIF-2α binding sites that are closest to transcribed loci of each RNA class (A). The distribution of (B) HIF-1α and (C) HIF-2α binding sites around the transcriptional start site of the nearest expressed gene irrespective of class (black bars). The grey bars show the distribution when only active mRNA genes are used (analogous to previous analyses).D–F GSEA analysis against fold-regulation by hypoxia for (D) all HIF-binding transcripts, (E) mRNA and (F) lncRNA.G Log2 fold-change of transcript abundance in hypoxia, ranked in order from highly upregulated to downregulated transcripts, from which the GSEA were derived.

As HIF is a major transcriptional regulator of the mRNA response to hypoxia 14, we next examined the role of HIF in the regulation of other classes of RNA. As a first step we examined for spatial association between each class of transcript and HIF-binding sites, using well validated, previously published HIF-1- and HIF-2-binding sites in MCF-7 cells 14. That work considered only protein coding genes and reported a non-random distribution of HIF binding across the genome. Although HIF-binding sites were strongly enriched at the promoters of coding genes, many HIF-binding sites were observed to be remote from these promoters. In the light of extensive regulation of non-coding RNAs by hypoxia, we reanalysed the distribution of HIF-binding in relation to the location of all transcripts. The number of HIF-binding transcripts (defined as the closest promoter to each HIF-binding site) in each category broadly mirrored the total number in each class. Although the numbers in many classes were too small to permit firm conclusions, no enrichment of HIF binding was observed in the vicinity of classes of RNA showing global downregulation (piwiRNAs, snRNAs and tRNAs) suggesting that they are unlikely to be directly regulated by HIF. In contrast, HIF binding was enriched in the vicinity of both mRNAs and lncRNAs (Fig2A) with HIF-2 binding a slightly higher proportion of lncRNAs than HIF-1. Nevertheless, despite promoter enrichment, the majority of HIF-1- and HIF-2-binding sites (50% and 70% respectively) were still found to lie in excess of 2.5-kb from the promoter of an expressed transcript of any class (Fig2B and C).

To assess the functional role of HIF in the regulation of different classes of RNA by hypoxia, we next examined for association of HIF binding with regulation by hypoxia across all classes of RNA using gene set enrichment analysis (GSEA) 15. All expressed transcripts were pooled and ranked according to their hypoxic induction. HIF binding was strongly enriched amongst transcripts upregulated (but not downregulated) by hypoxia (Fig2D). Only mRNA, lncRNA, miRNA and non-annotated transcripts were present in the core-enrichment group of HIF-binding hypoxia-inducible transcripts. GSEA analysis by subgroup confirmed enrichment for mRNAs (Fig2E) and lncRNAs (Fig2F), but the number of miRNAs and non-annotated RNAs was too small to permit statistical analysis. Taken together, this establishes a non-random spatial association between HIF-binding sites and hypoxia-inducible transcripts for both mRNAs and lncRNAs, pointing to a major new role for HIF in the transcriptional regulation of lncRNAs.

**Figure 3:**
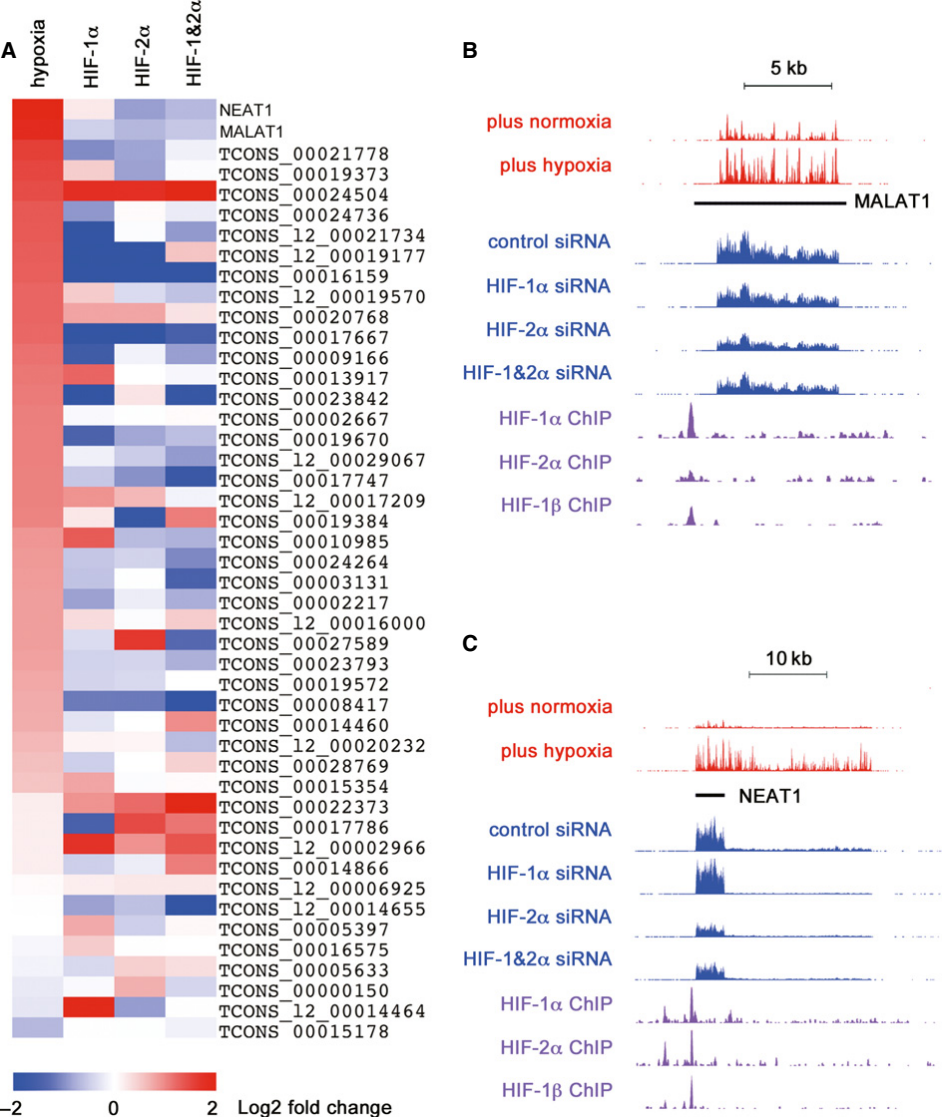
Direct transcriptional regulation oflncRNAs by HIF A Heat map showing fold regulation by hypoxia and by the indicated HIF siRNA for lncRNAs adjacent to HIF-binding site that are detected in the polyA RNA-seq analyses.B, C RNA-seq and HIF ChIP-seq genome browser tracks for the two most hypoxically upregulated HIF-binding lncRNAs: (B) MALAT1 and (C) NEAT1.

To pursue this further, we undertook polyA+ RNA-seq analysis of mRNA and lncRNA in hypoxic MCF-7 cells transfected with siRNAs targeting HIF-1α, HIF-2α or both isoforms simultaneously. Approximately 25% of all lncRNA transcripts detected in the ribosomal-depleted RNA-seq were detected in this analysis. Fig3A portrays the results of HIF-α siRNA interventions on all HIF-binding lncRNA transcripts detected in this dataset. As expected, the heatmap confirms that many of these RNAs were upregulated by hypoxia and reveals a strong correlation with down regulation by siRNAs targeting one or both HIF-α isoforms, a result that was confirmed by GSEA (supplementary Fig S7). Individual datasets are illustrated for MALAT1 (downregulated by both HIF-1α and HIF-2α siRNA, Fig3B) and NEAT1 (downregulated by HIF-2α but not by HIF-1α siRNA, Fig3C). Thus, the presence of HIF binding, hypoxic upregulation and subsequent downregulation by the suppression of HIF-α defines lncRNAs as a new group of HIF target genes.

### HIF-dependent recruitment and release of promoter-paused RNApol2 in hypoxia

To pursue the mechanism of transcriptional activation by hypoxia further, we next examined RNApol2 binding and histone H3K4me3 modification in normoxia and hypoxia. As expected, the promoter-associated abundance of each mark correlated with the basal transcript level. Strong peaks of RNApol2 and H3K4me3 signal were also seen at the putative transcriptional start sites (TSS) of the previously non-annotated transcripts consistent with assignment as *bona fide* genes (supplementary Fig S8) 16 17.

**Figure 4:**
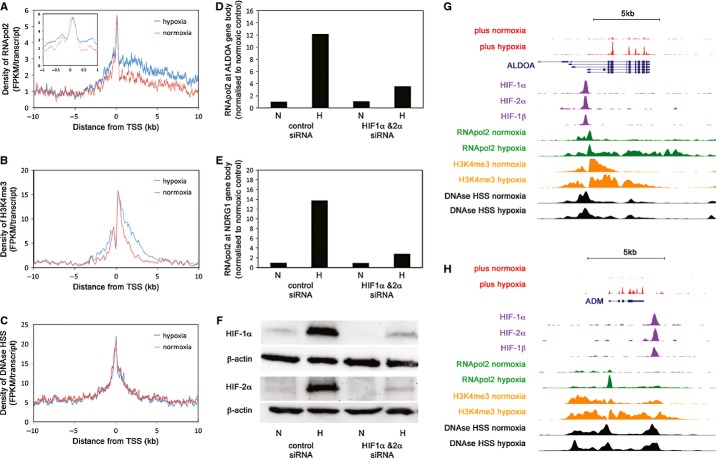
HIF predominantly regulates transcription through release of promoter-paused RNA-pol2 A–C Mean distribution of normoxic (red) and hypoxic (blue) RNApol2 binding at the 100 transcripts most upregulated by hypoxia. Inset shows expanded view at the TSS (FPKM = fragments per kilobase per million reads). The same plots for (B) H3K4me3 and (C) DNAse1 hypersensitivity are shown.D, E ChIP-qPCR analysis of RNApol2 within the body of (D) ALDOA and (E) NDRG1 gene shows suppression of the hypoxic induction by HIF-1α & HIF-2α siRNA (biological duplicates).F Immunoblot analysis of HIF-α levels.G,H RNA-seq and ChIP-seq tracks illustrating (G) release of promoter-paused RNApol2 and (H) *de novo* recruitment of RNApol2, despite constitutive DNAse1 hypersensitivity.

Consideration of changes in RNApol2 binding in hypoxia revealed that for the majority of hypoxia-inducible genes, RNApol2 was already bound at the promoter in normoxic cells. Although there were exceptions, at the majority of promoter sites hypoxia did not increase promoter-bound RNApol2 substantially. This is illustrated in Fig4A which depicts changes in RNApol2 ChIP signal in the vicinity of the promoters of the hundred most strongly hypoxia upregulated transcripts of any class. In hypoxia, little increase in loading of RNApol2 at the transcriptional start was seen; instead an increase in RNApol2 is observed across the body of the gene. Similar patterns of change in hypoxia were seen for both mRNAs and lncRNAs (supplementary Fig S9). Actively transcribing RNApol2 can recruit histone methyl transferase activity that trimethylates H3K4 either directly 18 or through p300 19. The distribution of H3K4me3 showed a very similar response to that of RNApol2, with little change in signal at the promoter in hypoxia, but an increase downstream of the transcriptional start site, consistent with the hypothesis that RNApol2 recruits histone methyl transferases that trimethylate H3K4 (Fig4B). In contrast, consideration of DNAse hypersensitivity analyses in normoxic and hypoxic MCF7 cells reported by the Encyclopedia of DNA Elements (ENCODE) consortium 17 revealed the expected hypersensitivity signals at these promoter regions, but did not reveal changes in hypoxia (Fig4C).

On close inspection the maximum RNApol2 signal was observed in the promoter-proximal region, 40–45 bp downstream of the transcriptional start. This suggests that promoter-proximal pausing is an important rate-limiting step for transcription of hypoxia-inducible genes and that reversal of pausing is a key step through which hypoxia regulates transcription 20. To determine whether this is a feature of direct transcriptional regulation by HIF, as opposed to indirect effects of HIF or hypoxia itself on transcription, we performed similar analysis of RNApol2 distribution for genes that were downregulated by hypoxia and which (consistent with indirect regulation) do not show promoter enrichment of HIF binding. We also compared hypoxia-inducible changes in patterns of RNApol2 binding and H3K4 trimethylation amongst groups of genes that were matched for the extent of upregulation by hypoxia, but were selected as being either the closest gene to a HIF-binding site (likely to represent direct transcriptional regulation) or were located >1 Mb from the nearest HIF-binding site (likely to represent indirect actions of HIF or hypoxia). Whilst a clear change in the ratio of promoter paused RNApol2 to that distributed along the gene body (travelling ratio) was observed for genes predicted to be direct HIF transcriptional targets this was not observed for genes predicted to be upregulated or downregulated indirectly by HIF or by hypoxia itself (supplementary Fig S10). Finally, ChIP-qPCR at a selection of these genes confirmed that the hypoxic induction of RNApol2 within the body of the gene was dependent on the presence of HIF-α subunits (Fig4D–F).

Amongst genes predicted to be direct transcriptional targets of HIF we observed a clear change in the distribution of travelling ratios in hypoxia, reflecting release of promoter-paused RNApol2 at most loci (Supplementary Fig S9E, F and Fig4G). However, we also observed infrequent HIF target genes where RNApol2 was recruited *de novo* in hypoxia (Fig4H). No association was detected between this type of behaviour and biological function. However, the promoters of these transcripts also show constitutive DNAse1 HSS signal indicating that other factors must limit normoxic RNApol2 binding at these sites. As DNA-seq cannot distinguish individual nucleosome positions, this might include mechanisms such as nucleosome positioning as well as DNA/histone methylation 21.

Taken together, our findings indicate that the regulation of gene expression by hypoxia is substantially more complex than has previously been considered. All RNA classes were regulated bi-directionally, our analysis identifying both general responses characteristic of particular types of RNA and high-amplitude effects on individual species. Both the non-random spatial association between HIF-binding sites and hypoxia-inducible genes and direct functional intervention by siRNA implicated HIF in the regulation of extensive networks of non-coding RNAs. RNApol2 profiling indicates that this direct regulation by HIF is mediated through activation of pre-bound RNApol2. These findings confirm and extend the role of HIF in the regulation of miRNAs and identify both intergenic and antisense lncRNAs as a new target class for the HIF transcriptional response. The ability of non-coding RNAs to regulate gene expression both in *trans* and in *cis* suggests the hitherto unrecognised involvement of HIF in a far broader network of gene regulation. Given the fundamental role of hypoxia in human pathology and emerging data on the importance of non-coding RNAs in cancer and other diseases, these findings open new avenues to the better understanding of human disease processes.

## Materials and Methods

### Cell culture & HIF siRNA treatment

Human MCF-7 breast carcinoma cells from the American Type Culture Collection (ATCC) were incubated for 24 h in an *In vivo2 Hypoxia Work Station* (Ruskinn Technology Ltd, Bridgend, UK) in an atmosphere of either normoxia (21% oxygen) or hypoxia (1% oxygen). Expression of HIF-1α and/or HIF-2α subunits was suppressed as previously described 22.

### Ribosomal depleted RNA-seq

Total RNA was prepared using the mirVana miRNA Isolation Kit (Ambion; Life Technologies Ltd, Paisley, UK) and treated with DNaseI (TURBO DNA-free™, Ambion). Ribosomal RNAs were depleted using RiboMinus™ Locked Nucleic Acid probes (Invitrogen; Life Technologies Ltd, Paisley, UK). The resulting RNA was fragmented and end-repaired using the Illumina directional protocol. The final cDNA library was purified using Agencourt AMPure beads (Beckman Coulter UK Ltd, High Wycombe, UK) optimised to retain small RNAs.

### PolyA+ selected RNA-seq

Directional PolyA+ RNA libraries were prepared using the ScriptSeq™ v2 RNA-Seq kit (Epicentre, Madison, WI, USA) and non-directional libraries using the TruSeqRNA Sample Prep Kit (Illumina, San Diego, CA, USA).

### ChIP-seq

Chromatin immunoprecipitation was performed as previously described 14. In addition to HIF ChIPs, antibodies to H3K4me3 (Cell Signalling, Danvers, MA, USA, #9751) and RNApol2 (Santa Cruz Biotechnology, Dallas, TX, USA, #sc-899) were used. Libraries were prepared using the Illumina ChIP-seq kit.

### High throughput sequencing

All libraries were sequenced using the GAII or HiSeq platforms (Illumina) according to the manufacturers protocol. Raw and mapped data for RNA-seq and ChIP-seq is available at EMBL-EBI Array Express (E-MTAB-1994, E-MTAB-1995).

### Bioinformatic analysis

After trimming adapter sequences using the FASTX-tool kit (http://hannonlab.cshl.edu/fastx_toolkit/), RNA-seq reads were sequentially mapped to RNA databases and expression analysis performed using the edgeR (Bioconductor, Seattle, WA, USA) package. Remaining unmapped reads were examined for non-annotated reads using the Cufflinks de novo transcript assembler (http://cufflinks.cbcb.umd.edu). ChIP-seq databases were analysed as previously described 14.

### Statistical analysis

All assays were performed in duplicate or triplicate. Statistical analyses using two-tailed *t*-tests were performed in SPSS (IBM Corp, New York, NY, USA) and R (http://www.R-project.org).

## Supporting Information

Supplementary Methods

Review Process File
