# Supplementary figures and images for: Extensive regulation of the non-coding transcriptome by hypoxia: role of HIF in releasing paused RNApol2
Source: EMBO Rep. 2013 Dec 22;15(1):70–6. doi: 10.1002/embr.201337642 (PMC3983684; doi:10.1002/embr.201337642)

Supplementary Figure 1

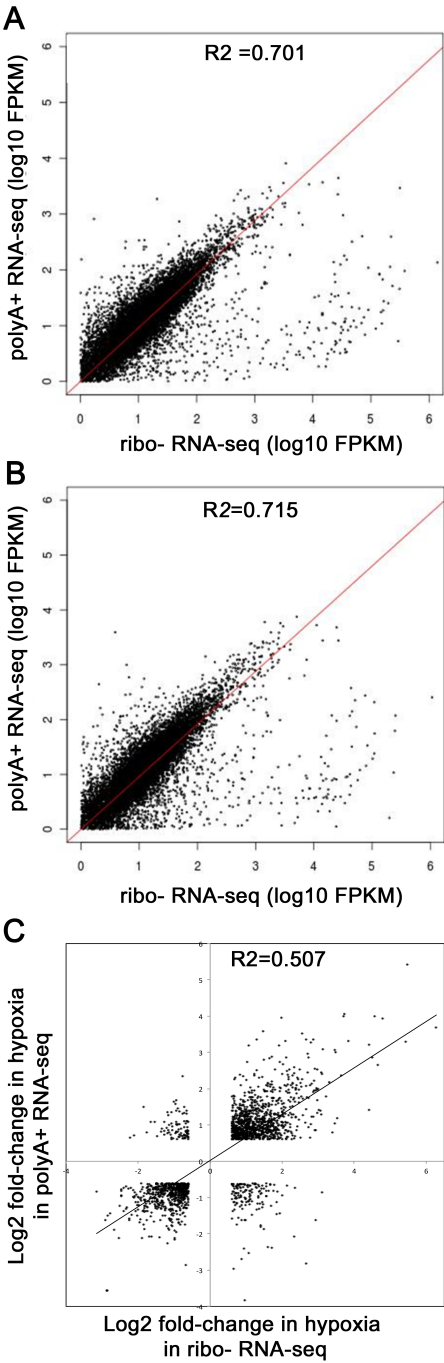

Supplement: Supplementary file 1 [file embr0015-0070-sd1.pdf]

## Supplementary Figure 2

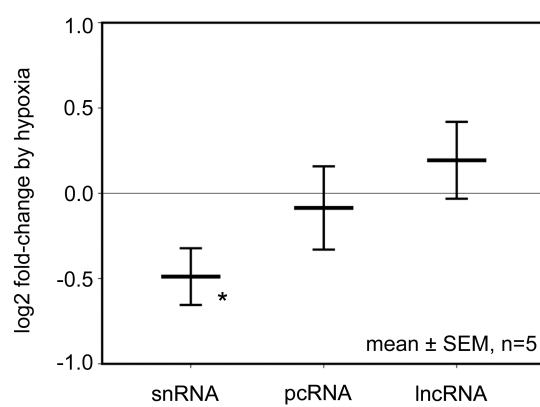

Supplement: Supplementary file 2 [file embr0015-0070-sd2.pdf]

### Supplementary Figure 3

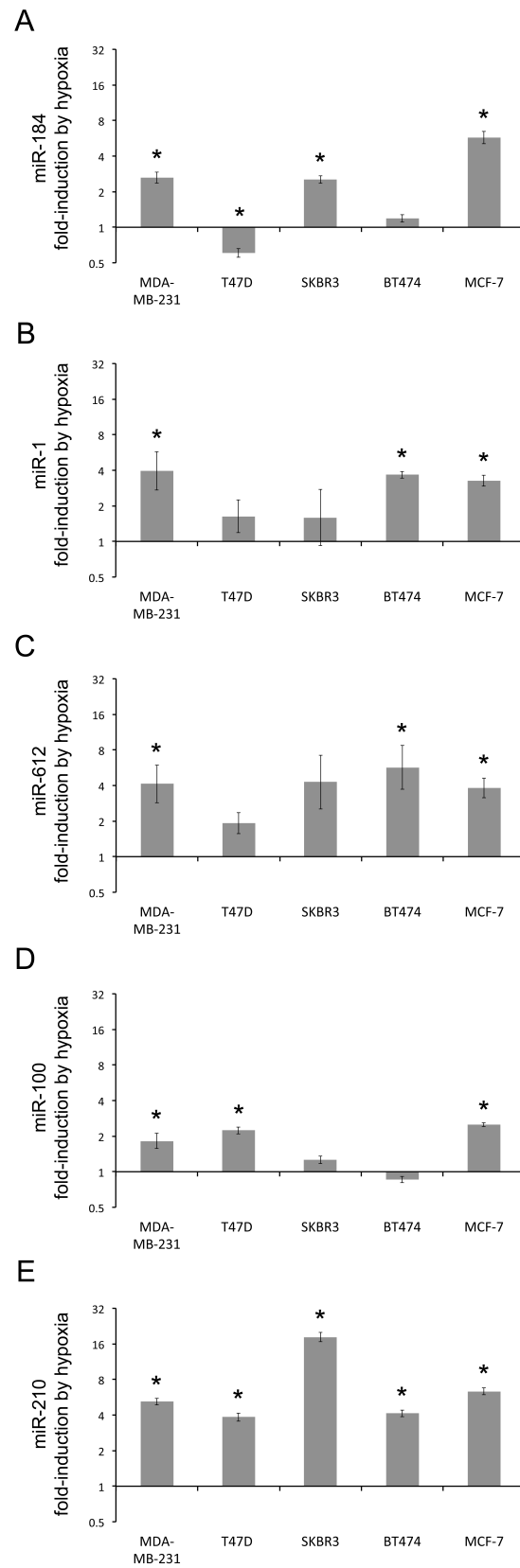

Supplement: Supplementary file 3 [file embr0015-0070-sd3.pdf]

Supplementary Figure 4

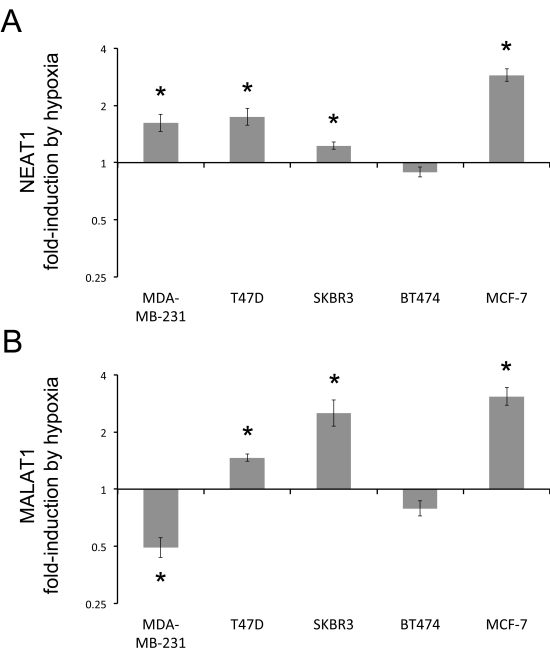

Supplement: Supplementary file 4 [file embr0015-0070-sd4.pdf]

Supplementary Figure 5

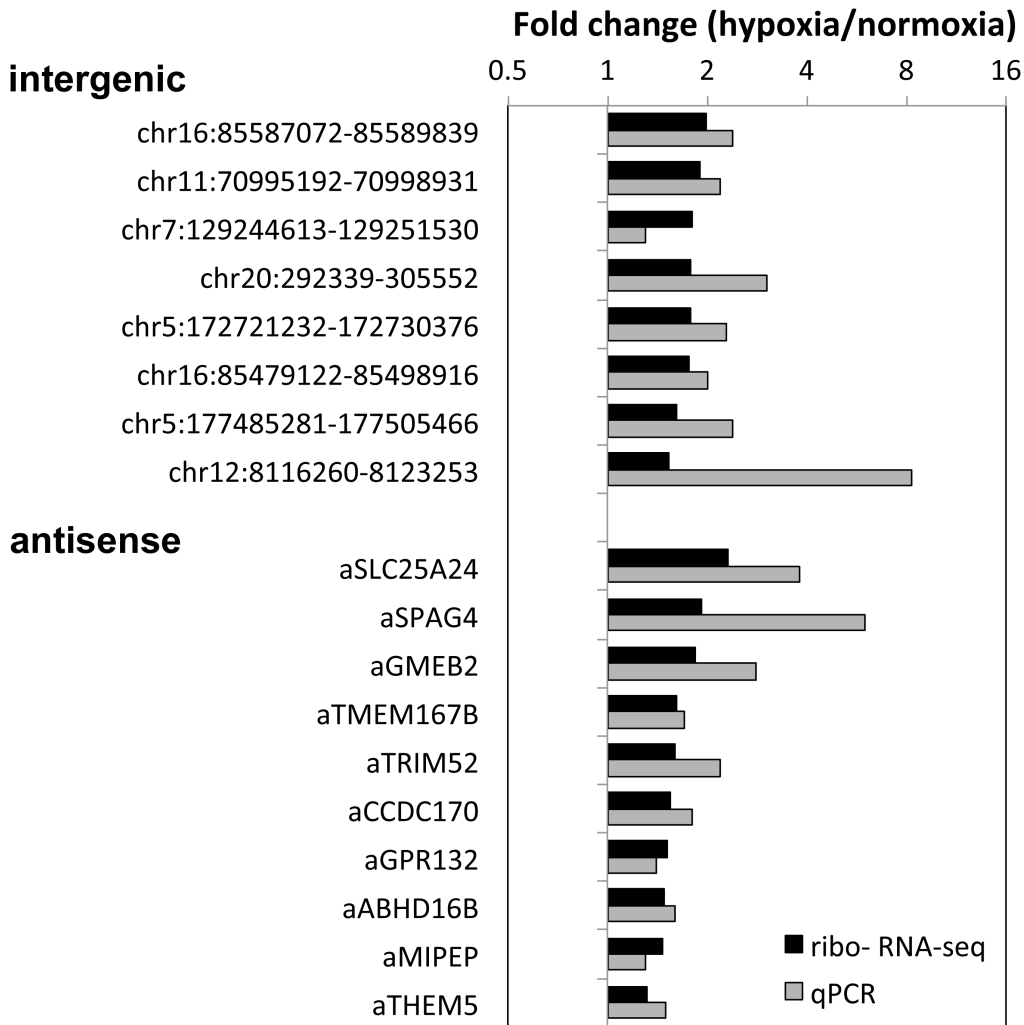

Supplement: Supplementary file 5 [file embr0015-0070-sd5.pdf]

## Supplementary Figure 6

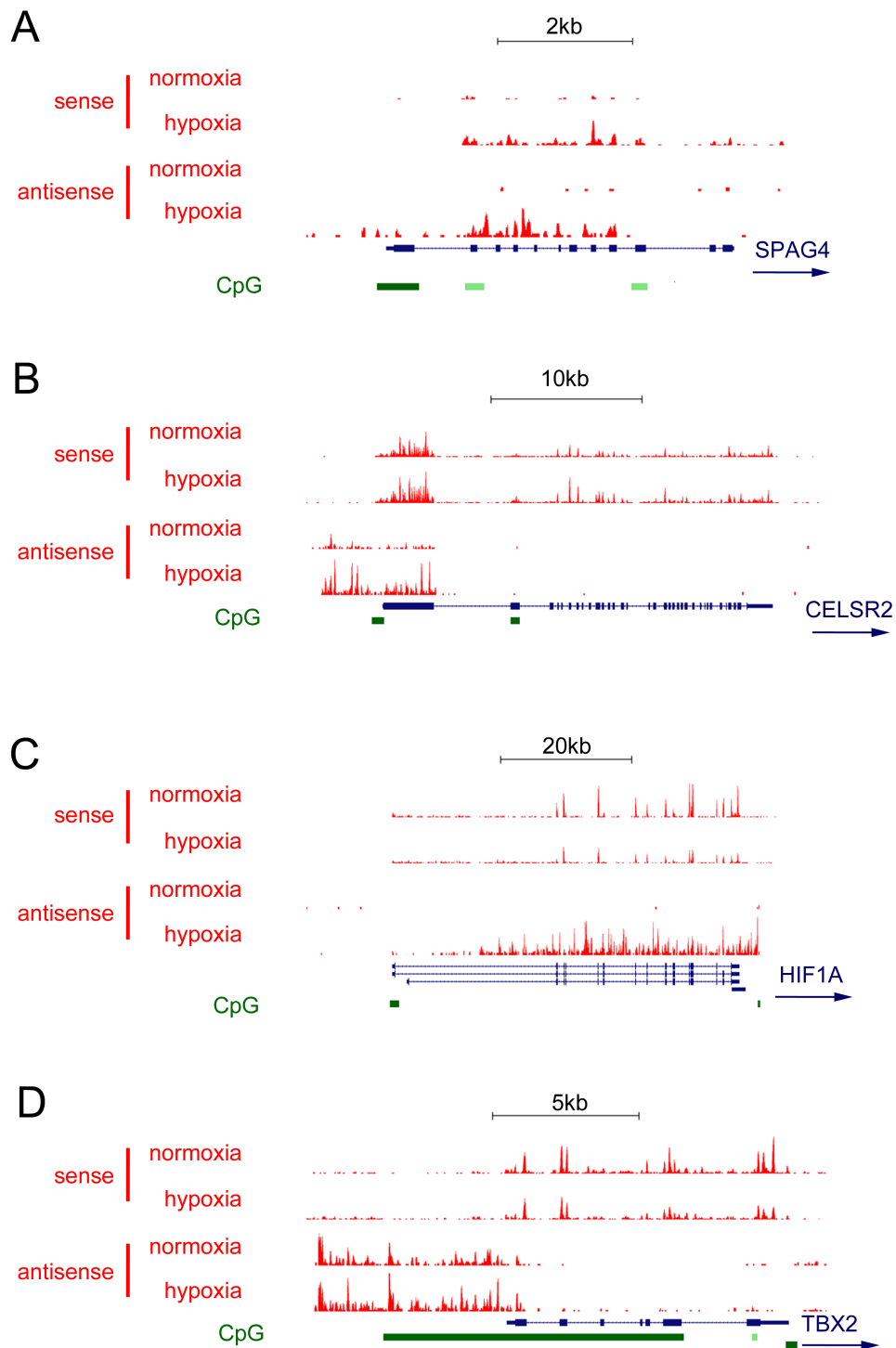

Supplement: Supplementary file 6 [file embr0015-0070-sd6.pdf]

Supplementary Figure 7

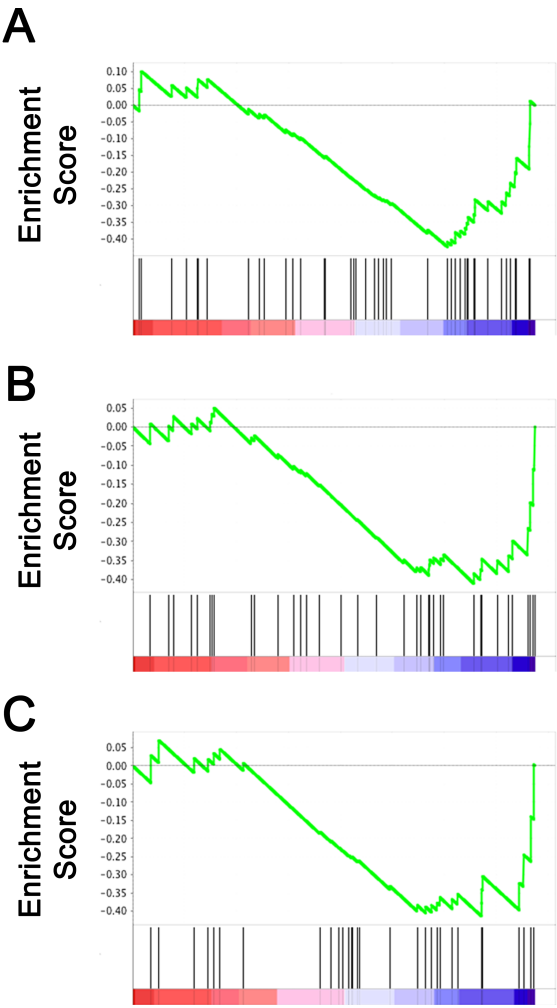

Supplement: Supplementary file 7 [file embr0015-0070-sd7.pdf]

**Supplementary Figure 8**

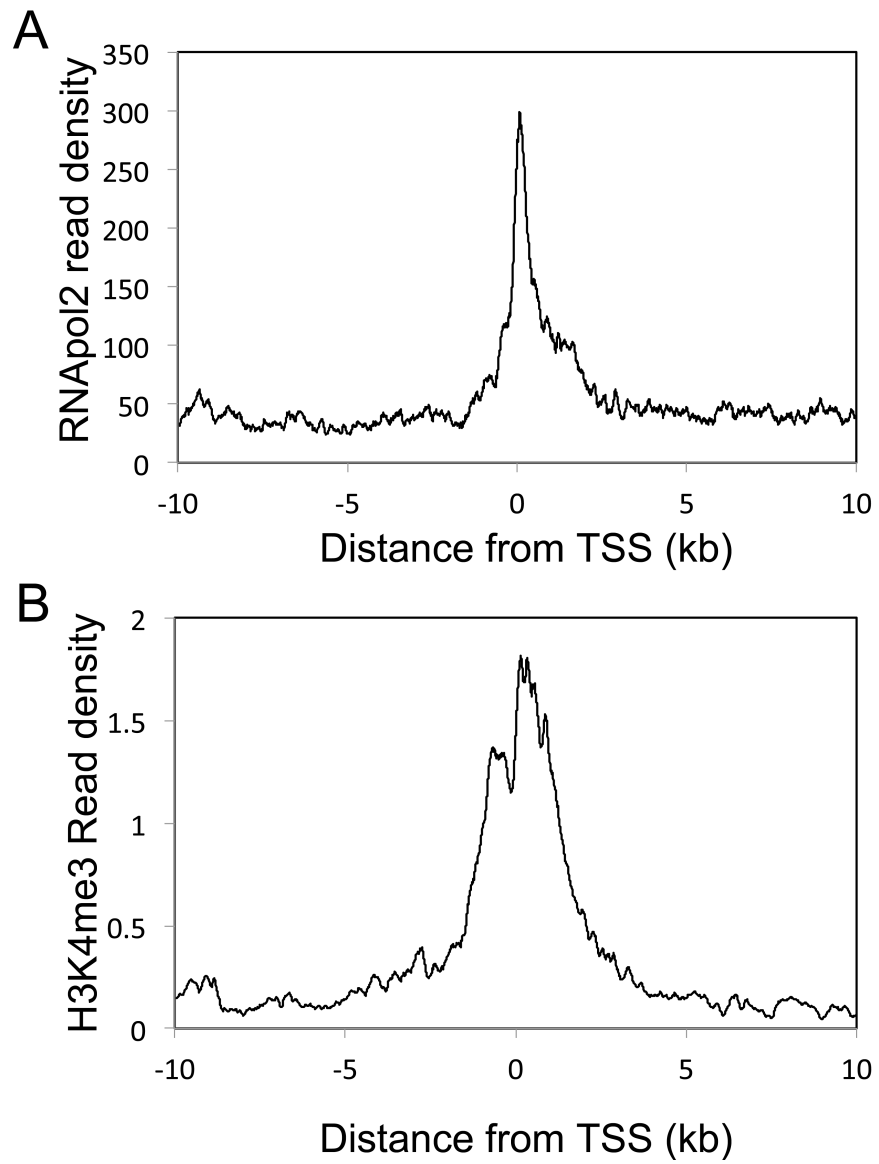

Supplement: Supplementary file 8 [file embr0015-0070-sd8.pdf]

## Supplementary Figure 9

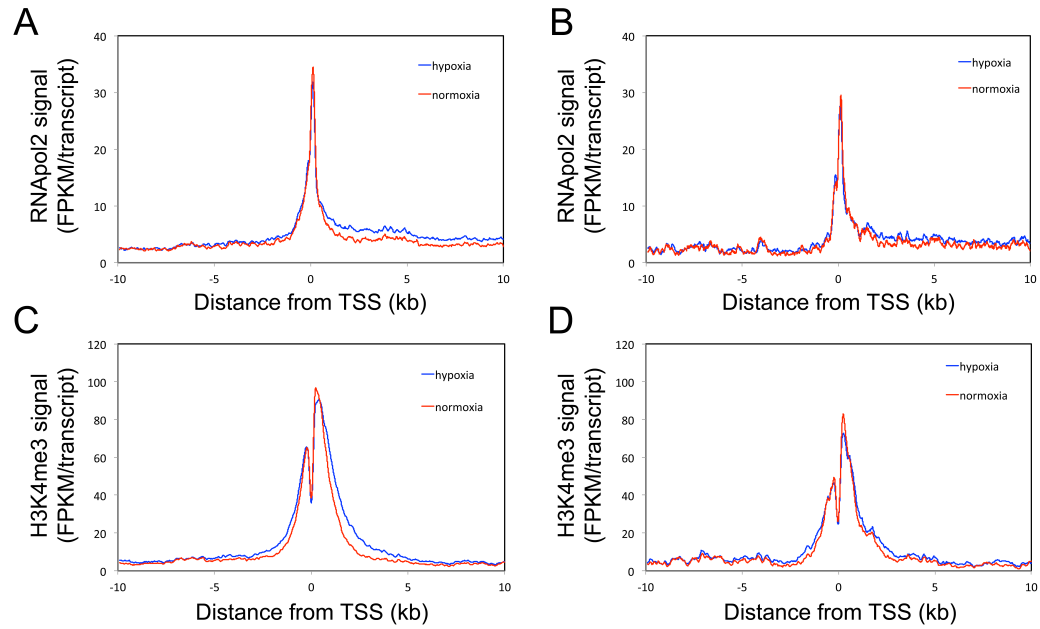

Supplement: Supplementary file 9 [file embr0015-0070-sd9.pdf]

**Supplementary Figure 10**

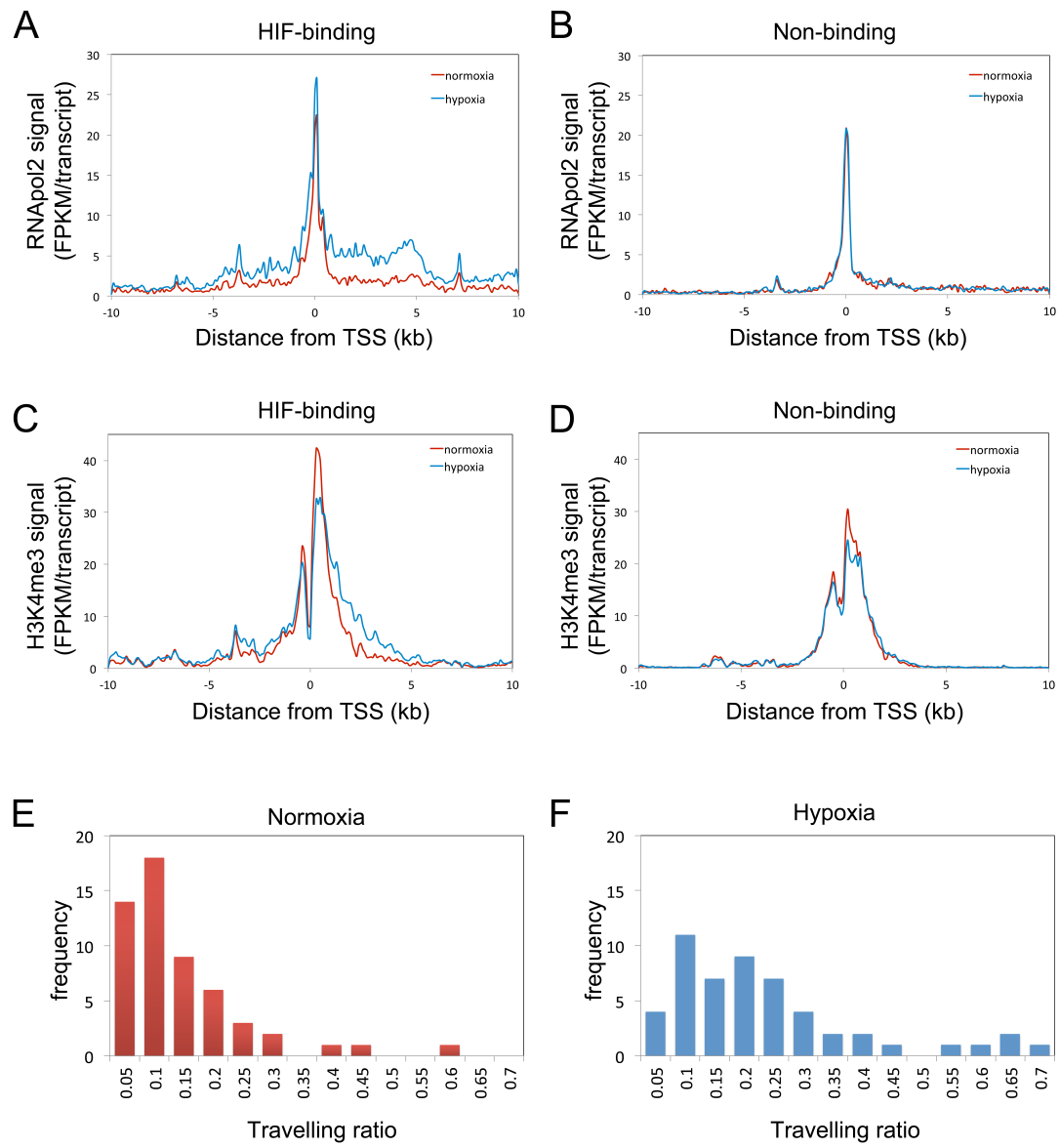

Supplement: Supplementary file 10 [file embr0015-0070-sd10.pdf]
