# Supplementary material for: Extensive regulation of the non-coding transcriptome by hypoxia: role of HIF in releasing paused RNApol2
Source: EMBO Rep. 2013 Dec 22;15(1):70–6. doi: 10.1002/embr.201337642 (PMC3983684; doi:10.1002/embr.201337642)
Supplement: Supplementary file 11 [file embr0015-0070-sd11.pdf]

Supplementary Table 1. Transcripts that manifest greatest fold-regulation in hypoxia for each class.

PiwiRNA					
**Genes**	**Chr**	**start**	**end**	**strand**	**Fold Change**
**Up regulated**					
piRNApiR-52416	chr1	229567886	229567917	-	14
piRNApiR-57604	chr6	74228271	74228299	-	4.9
piRNApiR-47161	chr1	228823233	228823260	+	4.9
piRNApiR-52681	chr17	17146601	17146629	+	4
piRNApiR-35058	chr14	90341397	90341425	-	2.5
**Down regulated**					
piRNApiR-32376	chr11	10530773	10530804	-	0.21
piRNApiR-36248	chr17	37023897	37023926	+	0.2
piRNApiR-31612	chr2	207026644	207026674	+	0.2
piRNApiR-49039	chr5	180633907	180633933	+	0.2
piRNApiR-36247	chr17	37023897	37023925	+	0.12

miRNA					
**Genes**	**Chr**	**start**	**end**	**strand**	**Fold Change**
**Up regulated**					
mir-184	chr15	79502130	79502213	+	205
mir-9-3	chr15	89911248	89911337	+	65
mir-9-1	chr1	156390133	156390221	-	64
mir-1-1	chr20	61151513	61151583	+	49
mir-1-2	chr18	19408965	19409049	-	49
**Down regulated**					
mir-539	chr14	101513658	101513735	+	0.35
mir-3614	chr17	54968631	54968716	-	0.3
mir-3658	chr1	165877158	165877213	+	0.18
mir-329-1	chr14	101493122	101493201	+	0.1
mir-620	chr12	116586365	116586459	-	0.03

snRNA/snoRNA					
**Genes**	**Chr**	**start**	**end**	**strand**	**Fold Change**
**Down regulated**					
ACA67B	chr2	10586839	10586974	-	0.1
ACA24	chr4	119200345	119200475	+	0.09
U31	chr11	62620796	62620866	-	0.08
U18C	chr15	66793589	66793655	-	0.06
SNORD124	chr17	38183794	38183897	-	0.04

tRNA					
**Genes**	**Chr**	**start**	**end**	**strand**	**Fold Change**
**Down regulated**					
tRNA114- Gln CTG	chr1	146476760	146476831	-	0.3
tRNA18- Tyr GTA	chr14	21125623	21125716	-	0.29
tRNA3- Asp GTC	chr3	184366095	184366165	-	0.29
tRNA2- Leu TAA	chr4	156384978	156385052	-	0.28
tRNA12- Tyr GTA	chr8	66609532	66609619	-	0.12

Protein coding RNA					
**Genes**	**Chr**	**start**	**end**	**strand**	**Fold Change**
**Up regulated**					
NDRG1	chr8	134249413	134309547	-	24
ALDOC	chr17	26900132	26903951	-	10
NR4A1	chr12	52445185	52453291	+	8.6
FOS	chr14	75745480	75748937	+	8
AHNAK2	chr14	105403590	105444694	-	7.6
**Down regulated**					
PCNA	chr20	5095598	5100647	-	0.23
HNRNPAB	chr5	177631507	177638184	+	0.23
PKIB	chr6	122973873	123047518	+	0.23
LTV1	chr6	144164507	144184943	+	0.22
CHAC2	chr2	53994928	54002287	+	0.18

lncRNA					
**Genes**	**Chr**	**start**	**end**	**strand**	**Fold Change**
**Up regulated**					
NEAT1	chr11	65190245	65192231	+	4.3
TCONS_00013573	chr7	123560905	123564184	+	4.2
TCONS_l2_00017632	chr22	22012077	22012712	+	4.2
TCONS_00015697	chr9	78194585	78203464	+	4.1
TCONS_00028106	chr20	10844845	10846396	+	4
**Down regulated**					
TCONS_00015820	chr9	46661444	46662837	-	0.29
TCONS_00007500	chr4	29119930	29204392	+	0.21
TCONS_l2_00023536	chr5	129096155	129100756	+	0.17
TCONS_00011521	chr6	79314184	79315753	-	0.12
TCONS_00011596	chr6	153115631	153153048	-	0.08
