# Supplementary material for: Extensive regulation of the non-coding transcriptome by hypoxia: role of HIF in releasing paused RNApol2
Source: EMBO Rep. 2013 Dec 22;15(1):70–6. doi: 10.1002/embr.201337642 (PMC3983684; doi:10.1002/embr.201337642)
Supplement: Supplementary file 12 [file embr0015-0070-sd12.pdf]

Supplementary Table 2 Comparison of hypoxia regulated miRNAs with previous reports

	Kulshreshtha	Hua	Hebert	Donker	Guimbellot	Choudhry
**Up regulated microRNAs**						
let-7b					↑	↑
let-7e					↑	↑
Let-7-i			↑			
miR-103	↑					↑
miR-106a	↑					
miR-107	↑					↑
miR-125a				↑	↑	↓
miR-125b	↑					
miR-128a					↑	
miR-137					↑	
miR-148a			↑		↑	
miR-148b			↑			
miR-151		↑				↑
miR-152				↑		
miR-15a			↑			↓
miR-181a	↑					
miR-181c	↑					
miR-185					↑	
miR-188		↑		↑		
miR-191			↑	↑		↑
miR-192	↑					
miR-193b				↑		↑
miR-199a					↑	
miR-20					↑	
miR-200a			↑			
miR-200b				↑		↓
miR-204					↑	
miR-206				↑		
miR-21	↑					
miR-210	↑	↑	↑	↑	↑	↑
miR-213	↑			↑	↑	
miR-214			↑		↑	
miR-23a	↑			↑		
miR-23b	↑			↑	↑	

miR-24-1	↑					↑
miR-26a	↑				↑	
miR-27a	↑					↑
miR-27b				↑		↑
miR-299					↑	
miR-30a-3p					↑	
miR-30a-5p				↑		
miR-30c				↑	↑	
miR-30d		↑		↑		↑
miR-335					↑	
miR-339				↑		↑
miR-342					↑	↓
miR-373			↑			
miR-429			↑			
miR-452*				↑		
miR-491				↑		
miR-498			↑			
miR-512-5p				↑		
miR-563			↑			
miR-572			↑			
miR-628			↑			
miR-637			↑			
miR-7			↑			
miR-93	↑			↑		
miR-98			↑			↑
**Contrasting regulation**						
Let-7-e		↓	↑			
Let-7-g		↓	↑			
miR-150				↓	↑	
miR-155		↑		↓	↑	
miR-16		↓			↑	
miR-181b	↑	↑		↓	↑	
miR-195	↑		↓			
miR-26b	↑	↓			↑	
miR-30b	↑	↓	↑		↑	
**Down regulated microRNAs**						
Let-7-f		↓				
miR-29b			↓			
miR-30e-5p			↓			

miR-373*				↓		
miR-374			↓			
miR-422b			↓			
Let-7-a		↓				
Let-7-c		↓				
Let-7-d		↓				
miR-101			↓			
miR-122a			↓			
miR-128b				↓		
miR-141			↓			↓
miR-15b		↓				
miR-181d				↓		↑
miR-186			↓			
miR-196a				↓		
miR-196b				↓		
miR-197			↓			
miR-19a			↓			
miR-200a*				↓		
miR-20a		↓				
miR-20b		↓				
miR-216					↓	
miR-224		↓				
miR-25				↓		
miR-320			↓			
miR-424			↓	↓		
miR-449				↓		
miR-519e*				↓		
miR-565			↓			
miR-9					↓	
miR-92				↓		
miR-489				↓		

Studies: **Kulshreshtha et al[1]** - colon and breast cancer cells, 0.2% O_2, 8–48 h; **Hua et al[2]** - nasopharyngeal carcinoma cells, DFOM treatment, 20 h; **Hebert et al[3]** - head and neck squamous carcinoma cells, 1% O_2, 1 h or 5% O_2, 8 h; **Donker et al[4]** - primary human cytotrophoblasts, 1% O_2, 48 h; **Guimbellot et al[5]** - colon cells, liquid–liquid interface.
