# Supplementary material for: Extensive regulation of the non-coding transcriptome by hypoxia: role of HIF in releasing paused RNApol2
Source: EMBO Rep. 2013 Dec 22;15(1):70–6. doi: 10.1002/embr.201337642 (PMC3983684; doi:10.1002/embr.201337642)
Supplement: Supplementary file 13 [file embr0015-0070-sd13.pdf]

Supplementary Table 3. Non-annotated intergenic transcripts

ID	Chr	Start	End	Strand	Fold change in hypoxia	CPC
Novel 1	chr1	202053848	202082554	+	1.5	-0.63
Novel 2	chr1	202053658	202057903	+	1.2	-0.6
Novel 3	chr1	202002600	202088108	+	1.6	-0.56
Novel 4	chr1	201983168	202077686	+	1.6	0.84
Novel 5	chr1	181069443	181074240	-	1.5	-0.95
Novel 6	chr1	148241355	148241861	+	1.4	-1.32
Novel 7	chr1	107292908	107293420	+	1.2	-1.04
Novel 8	chr1	107073060	107424139	+	1.1	0.73
Novel 9	chr1	106734807	106876406	+	0	-0.24
Novel 10	chr1	106735450	106762265	+	1.1	-0.77
Novel 11	chr1	45196573	45196842	-	0.3	-1.36
Novel 12	chr10	8911651	8932114	+	1.1	-0.99
Novel 13	chr10	8909994	8931788	+	1.3	0.26
Novel 14	chr10	5524638	5535702	-	1.4	-0.52
Novel 15	chr11	65222490	65244737	+	1.7	-0.84
Novel 16	chr12	25956136	25959121	+	0.8	0.41
Novel 17	chr12	8116260	8123253	-	1.5	-0.81
Novel 18	chr15	51328348	51690847	+	1.4	-0.78
Novel 19	chr16	85587072	85589839	-	2	-0.88
Novel 20	chr16	85495495	85603598	+	1.7	0.005
Novel 21	chr16	85479122	85498916	-	1.8	-0.77
Novel 22	chr16	14395080	14494860	+	1.5	-0.57
Novel 23	chr16	85496110	85524891	+	1.6	-0.46
Novel 24	chr2	132300855	132350688	+	1.5	-0.58
Novel 25	chr2	75136093	75145898	+	1.3	-0.86
Novel 26	chr2	1571357	1629598	-	1.5	-0.34
Novel 27	chr20	292339	305552	-	1.8	-0.87
Novel 28	chr3	64022004	64104438	+	1.3	-0.79
Novel 29	chr5	177485281	177505466	-	1.6	-0.73
Novel 30	chr5	172721232	172730376	+	1.8	0.43
Novel 31	chr5	102088692	102198533	+	1.4	-0.86
Novel 32	chr5	66509857	66520819	+	1.4	-0.87
Novel 33	chr5	66509437	66891986	+	1.2	0.75
Novel 34	chr7	157283010	157312766	+	1.4	-0.27
Novel 35	chrX	95228382	95229237	-	1.3	0.02
Novel 36	chr11	70995192	70998931	+	1.8	-0.52
Novel 37	chr7	129244613	129251530	-	1.9	-0.99
