# Supplementary material for: Extensive regulation of the non-coding transcriptome by hypoxia: role of HIF in releasing paused RNApol2
Source: EMBO Rep. 2013 Dec 22;15(1):70–6. doi: 10.1002/embr.201337642 (PMC3983684; doi:10.1002/embr.201337642)
Supplement: Supplementary file 14 [file embr0015-0070-sd14.pdf]

Supplementary Table 4. Non-annotated anti-sense transcripts (NATs)

Name	Chr	Start	End	Strand	Fold regulation	Overlapping region of original gene	Regulation of sense gene
aARMC5	chr16	31461767	31470706	-	1.6	5'	Up
aATF7IP2	chr16	10527741	10712970	-	1.5	3'	Up
aMAMDC4/ PHPT1	chr9	139738901	139759957	-	1.3	Cover	Up
aTSEN54	chr17	73517363	73521618	-	1.3	3'	Down
aTPBG	chr6	83072075	83075226	-	1.3	5'	Up
aTBX2	chr17	59470718	59477783	-	1.5	5'	Down
aSTXBP2	chr19	7697325	7702009	-	1.6	5'	Up
aSPAG4	chr20	34196650	34207240	-	1.4	5'	Up
aCHRAC1	chr8	141516112	141521724	-	1.5	5'	Down
aGRIN1	chr9	140056711	140062733	-	1.5	3'	Not expressed
aHIF1A	chr14	62175449	62214842	-	1.3	3'	Down
aLENG8	chr19	54959291	54960259	-	1.3	5'	Up
aLOC338758	chr12	90056440	90103653	-	1.4	5'	Up
aSPTSSB	chr3	161089733	161146159	+	1.2	5'	Up
aBRAT1	chr7	2577658	2587164	+	1.1	3'	Down
aZIC4	chr3	147111164	147128972	+	1.6	5'	Non regulated
aTUT1	chr11	62341769	62346553	+	1.3	Middle	Non regulated
aTRIM17	chr1	228594940	228602400	+	1.3	3'	Down
aTHEM5	chr1	151812700	151825963	+	1.3	3'	Not expressed
aTBC1D10B	chr16	30366581	30370364	+	1.5	3'	Up
aSRMS	chr20	62170196	62175250	+	1.3	3'	Up
aSLC39A4	chr8	145638718	145644681	+	1.5	5'	Non regulated
aRASEF	chr9	85677150	85684988	+	1.1	5'	Up
aPTPRN	chr2	220163641	220168902	+	1.3	3'	Not expressed
aPRICKLE2	chr3	64021527	64102071	+	1.1	5'	Up
aCPLX1	chr4	776511	783395	+	1.4	3'	Non regulated
aGMEB2	chr20	62258636	62259915	+	1.3	5'	Up
aGPR132	chr14	105499226	105522613	+	1.5	3'	Up
aHDLBP	chr2	242157867	242184464	+	1.8	3'	Not expressed
aKPNA1	chr3	122135105	122146064	+	1.5	3'	Down
aLCAT	chr16	67969845	67982503	+	1.4	Cover	Non regulated
aBCAS3	chr17	59432883	59480160	-	1.5	3'	Down
aCELSR2	chr1	109788560	109796206	-	1.5	5'	UP
aBCAN	chr1	156607605	156616958	-	1.4	5'	Up regulated
aADRM1	chr20	60880596	60884025	-	1.1	Middle	Down
aAPC2	chr19	1457662	1461617	-	1.1	Middle	not expressed
aPCIF1	chr20	44562017	44563625	-	1.4	5'	Up
