# Supplementary material for: Extensive regulation of the non-coding transcriptome by hypoxia: role of HIF in releasing paused RNApol2
Source: EMBO Rep. 2013 Dec 22;15(1):70–6. doi: 10.1002/embr.201337642 (PMC3983684; doi:10.1002/embr.201337642)
Supplement: Supplementary file 15 [file embr0015-0070-sd15.pdf]

**Supplementary Table 5.** Oligonucleotides for qPCR

| ID | Forward Primer 5'-3' | Reverse Primer 5'-3' |
|---|---|---|
| NEAT1 | CCAGTTTTCCGAGAACCAAA | ATGCTGATCTGCTGCGTATG |
| MALAT1 | GGAAAGCGAGTGGTTGGTAA | TCTCCAGGACTTGGCAGTCT |
| aSPAG4 | CCTCCAAAGAGCTGTTCCTG | CTAGCCGACCCTCTCTTCCT |
| aSLC25A24 | GTGTCTGCCAGCTGCACTTA | GACTACCCGGAGACGCTATG |
| aGMEB2 | GACGCTGCTGTGTGTGAATG | GCCCTCCTCCTACAAGTGTG |
| aTMEM167B | AGAAGCCGTAGTACGCAGGA | AGTTACCACGGCCAAGTACG |
| aTRIM52 | CTAAAGCTGAAGGGCAGGTG | TTTCAAAACTGTGGGTGTGC |
| aCCDC170 | TAGTCTTGCGCTTCCTGACA | CAGCTGTTTACCGTGTAGGTCA |
| aGPR132 | GACCTGGGAGAACAGTTCCA | TCCAGCCTCTAGCCACAGTT |
| aABHD16B | CGGGGACTAGGGACATGTAG | CCTAGCTCTGTCCCCTCCTT |
| aMIPEP | GGTTGAGAGGTCCTGTGCTC | AGCCTTTGTCCTGAGTTCCA |
| aTHEM5 | GTTGCCAAGCATGTGACTGT | TTGGATCCTCCACAGACTCC |
| chr16:85587072-85589839 | GGTCGGAGAGAGAGCCTTTT | TTCCATAGTGGCCAGAGAGG |
| chr11:70995192-70998931 | TGCTGTGCACTTGTGTGTTC | CCCCTGCAGTCACCTTTTAT |
| chr7:129244613-129251530 | TGAAGGAGTAGGAGGGCAAA | AAACACGTGGCTTTCCTGTT |
| chr20:292339-305552 | GACCAGCATCTCAACTGCAA | TCTTCCCCTCCTCTTCCAAT |
| chr5:172721232-172730376 | CGCAACTGTAGCACATGGAT | TGCCTTCGCAAGAGTCTTCT |
| chr5:177485281-177505466 | GTAGGGCCTGTGCCTTTTCT | TGGTTGAGACGTCTGTGAGC |
| chr12:8116260-8123253 | AAAAAGGAGAGCGCGTACTG | GCCCGAACTACAATTCTTGG |
| chr16:85479122-85498916 | ACCACCTAAGCCGACATCTG | CTCTGTGTGCACCTCTCTGC |
| ALDOA gene body | GCTATGGCCTTTTCCTTTCC | AACTCTGTCTGGTGCTGGGT |
| NDRG1 gene body | GAAGTCACAGGTCAGGGGAA | AGGGAGAGAGAGGGTTTTGG |
