# Supplementary material for: Extensive regulation of the non-coding transcriptome by hypoxia: role of HIF in releasing paused RNApol2
Source: EMBO Rep. 2013 Dec 22;15(1):70–6. doi: 10.1002/embr.201337642 (PMC3983684; doi:10.1002/embr.201337642)
Supplement: Supplementary file 16 [file embr0015-0070-sd16.pdf]

Supplementary Information:

Supplementary Figure Legends

Supplementary Figure 1. Correlation between polyA+ and ribosomal depleted-RNA-seq. Using transcripts present in both datasets, the expression level assessed by polyA+ RNA-seq was plotted against the transcript level in the ribo- RNA-seq dataset for (A) normoxic and (B) hypoxic samples. Linear regression shows a high degree of correlation between the two types of analysis. (C) Transcripts were then filtered for those showing at least 1.5 fold regulation by hypoxia in both the polyA+ and ribo-datasets and the two values plotted against each other. Log2 fold-change in hypoxia again correlated well between the two types of analysis.

Supplementary Figure 2. qPCR validation of global changes in transcript abundance during hypoxia. For each of the RNA classes shown, five transcripts exhibiting hypoxic regulation close to the median value were assayed by TaqMan gene expression assays. The five snRNAs showed significant down regulation, whilst the mean changes for the five protein-coding RNAs and the five lncRNAs did not show significant change (* $p<0.05$, student 1-sample t-test). All the experiments were performed with three biological replicates for each of the five (n=5) transcripts in each class. snRNAs: SNO38, SNO117, SNO60, SNO21, SNO66. mRNAs: TCF25, C15orf42, DHX30, ZNF320, RBAK. LncRNAs: TCONS_l2_00027397, TCONS_00019713, TCONS_l2_00016774, TCONS_00020202, TCONS_00000020.

Supplementary figure 3. Hypoxic regulation of miRNAs in a panel of breast cancer cell lines. qPCR analysis of hypoxic regulation of (A) miR-184, (B) miR-1, (C) miR-612, (D) miR-100 and (E) miR-210 in the designated cell lines. Bars represent fold-change after 24 hours in 1% hypoxia ± SEM, n = 3. (* $p < 0.02$, Student's t-test)

Supplementary Figure 4. Hypoxic regulation of NEAT1 and MALAT1 in a panel of breast cancer cell lines. qPCR analysis of hypoxic regulation of (A) NEAT1 and (B) MALAT1 in the designated cell lines. Bars represent fold-change after 24 hours in 1% hypoxia ± SEM, n = 3. (* $p < 0.02$, Student's t-test).

Supplementary Figure 5. qPCR validation of non-annotated intergenic and antisense transcripts regulated by hypoxia. Black bars show fold-regulation in ribo- RNA-seq dataset and grey bars show fold-regulation by qPCR for the most upregulated transcripts of each class. All the experiments were performed in three biological replicates. "a" before gene symbol indicates anti-sense transcript of the gene.

Supplementary Figure 6. Co- and counter-regulation of overlapping sense and antisense transcripts. Ribosomal depleted directional RNA-seq tracks illustrating co-regulation of sense and antisense transcripts at (A) sperm associated antigen 4 (SPAG4) (B) cadherin, EGF LAG seven-pass G-type receptor 2 (CELSR2), and

counter-regulation at (C) HIF-1α and (D) T-box 2 (TBX2). The arrow under each gene symbol represents the strand from which each RNA is transcribed.

Supplementary Figure 7. lncRNAs adjacent to HIF binding sites are regulated by HIF. (A) GSEA analysis showing enrichment of lncRNAs adjacent to HIF-binding sites amongst those down regulated by combined HIF-1α and HIF-2α siRNA in hypoxia. The same analysis was repeated for (B) HIF-1α siRNA and (C) HIF-2α siRNA alone.

Supplementary Figure 8. RNApol2 and H3K4me3 marks at putative TSS of non annotated transcripts. ChIP-seq signals for the promoter associated marks, (A) RNApol2 and (B) H3K4me3 at the putative TSS of non-annotated transcripts.

Supplementary Figure 9. Hypoxic regulation of RNApol2 and H3K4me3 at pcRNAs and lncRNAs gene loci. Normoxic (red) and hypoxic (blue) signal for (A) RNApol2 and (C) H3K4me3 averaged over the 100 most hypoxically up-regulated pcRNA gene loci. (B & D) The same plots for the 100 most up-regulated lncRNAs. FPKM = fragments per kilobase per million reads.

Supplementary Figure 10. Hypoxic regulation of RNApol2 and H3K4me3 at HIF-binding and non-binding gene loci upregulated by hypoxia. pcRNAs upregulated by hypoxia were classified according to HIF-binding status. Transcripts

closest to and within 100 kb of a high-stringency HIF binding site were defined as HIF-binding. Transcripts with no discernable HIF ChIP-seq signal within 1 Mb of the promoter were defined as non-binding. Matched pairs of up-regulated transcripts from each group were selected to have equivalent fold-regulation. Shown are normoxic (red) and hypoxic (blue) signal for (A) RNApol2 and (C) H3K4me3 averaged over HIF-binding transcripts and (B and D) the same plots for non-binding loci. (E) Normoxic and (F) hypoxic RNApol2 travelling ratio (number of reads across the body of the gene / number of reads at promoter (-150bp to +250bp)) for the most upregulated HIF-binding transcripts. FPKM = fragments per kilobase per million reads.
