# Supplementary material for: Extensive regulation of the non-coding transcriptome by hypoxia: role of HIF in releasing paused RNApol2
Source: EMBO Rep. 2013 Dec 22;15(1):70–6. doi: 10.1002/embr.201337642 (PMC3983684; doi:10.1002/embr.201337642)
Supplement: Supplementary file 17 [file embr0015-0070-sd17.pdf]

Supplementary Methods

Cell Culture

Additional cell lines (SKBR3, T47D, BT474 and MDA-MB-231) were obtained and cultured according to the American Type Culture Collection (ATCC). Hypoxic incubation was as for MCF-7 cells and is described in the main methods.

Ribosomally depleted directional RNA-seq (ribo- RNA-seq)

Total RNA was isolated from MCF-7 cells grown in normoxia (21% O_2) or in hypoxia (1% O_2) for 24 hours. After DNAse treatment (Ambion, Inc), the rRNA fraction was depleted by hybridization with Locked Nucleic Acid (LNA) probes (RiboMinus™, Invitrogen). rRNA depletion was assessed using the Agilent RNA 6000 PicoKit (Agilent Technologies). The cDNA library was created using a modified Illumina directional RNA-seq protocol. Briefly, ribosomal-depleted RNA was fragmented by heating at 94 °C for 8 min in 1× fragmentation buffer (Affymetrix). Reactions were terminated by the addition of EDTA to a final concentration of 167 mM and cooling to 4 ºC. End repair of fragmented RNA, adapter ligation, cDNA synthesis and library amplification were performed according the standard Illumina directional RNA-seq protocol. The final cDNA library was purified using AMPure beads according to a protocol optimised to include small RNAs. Levels of rRNA contamination were assessed by cloning the library product into Zero Blunt TOPO vector (Invitrogen) and Sanger sequencing of representative bacterial colonies.

Normoxic and hypoxic libraries were sequenced on the Illumina *Genome Analyzer* II (GXII) platform.

Bioinformatic analysis

Mapping pipeline:

RNA-seq data were mapped using *in-house* analysis pipelines. These allowed sequential mapping of the raw data to specific RNA databases and for calculation of the expression level of the mapped transcripts. The analysis pipeline was initiated by trimming the adapter sequences from the raw sequence data using the FASTX-tool kit (http://hannonlab.cshl.edu/fastx_toolkit/). The sequences were mapped sequentially to databases of different RNA classes, according to Figure 1B, using Bowtie (version 1.0.0)[6]. The RNA databases used were: human protein coding database (UCSC), rRNA database (Wellcome Trust Centre for Human Genetics bioinformatics group), piwiRNA database (NCBI), microRNA database (mirBase), sn/snoRNA database (UCSC), tRNA database (UCSC), and long coding RNAs (ENCODE). All databases were based on the 2009 human genome release, hg19. The first round of mapping used stringent criteria (1 mismatch, threshold of mismatches ≤ 70) and was followed by a second less stringent round (2 mismatches).

Analysis of non-annotated transcripts:

We next used the Cufflinks package (version 2.1.1)[7], with default settings, to *de novo* assemble transcripts from contiguous reads that had not mapped to the annotated databases above. We excluded transcripts with an abundance of less than 13% of neighbouring annotated transcripts or that overlapped an annotated transcript from the

same orientation. Transcripts were also filtered for the presence both RNAPol2 and H3K4me3 at their putative TSS. The remaining filtered transcripts were defined as intergenic (no overlap with annotated transcripts) or anti-sense (overlapping with but in the opposite orientation to an annotated transcript). We further required that anti-sense transcripts overlapped the "sense" transcript by at least 100 reads. To assess the coding potential of transcripts ''CPC calculator '' tool was used[8].

Quantification of gene expression:

To determine transcript regulation, mapped sequence data was normalised to the total number of mapped reads (excluding the ribosomal reads). Minimum thresholds were then applied to transcript abundance (10 reads per transcript piwiRNA, miRNA, snRNA and tRNA and 100 reads per transcript for pcRNAs and lncRNAs), since at low abundance, the estimation of hypoxic regulation was poor (Figure 1C). the ratio of normalised reads between to samples was calculated as fold change.

ChIP-seq and peak calling analysis:

All ChIP-seq data sets were mapped using Bowtie (version 1.0.0) [6] to the human genome (build GRCh37/hg19). Data were normalised to the total number of mapped reads in each dataset. Peak finding was performed using CisGenome as previously described[9].

Quantification of miRNA expression

Total RNA was reverse transcribed using the TaqMan® MicroRNA Reverse Transcription Kit (Applied Biosystems, USA). miRNA expression levels were determined using TaqMan MicroRNA Analysis Assays (Applied Biosystems assay IDs: has-miR-612;001579, has-miR-184;000485, has-miR-210;000512, has-miR-100;000437, has-miR-1;002222; RNU43;001095). Expression levels were normalised to RNU43 housekeeping gene. qPCR was performed using the CFX96 real-time PCR detection system (BioRad, Hercules, California, US)

Quantitative PCR (qPCR) and TaqMan assay:

Reverse transcription for cDNA synthesis was performed using 500ng of RNA using SuperScript II Reverse Transcriptase (Invitrogen). qPCR was performed using IQ SYBR Green Mix (Bio-Rad) or TaqMan Universal PCR Master Mix (Applied Biosystems) on the on the CFX96 Real-Time System (Bio-Rad). Results were normalised to expression of *RPL11 (60S ribosomal protein L11)* for both assays. All experiments were performed using three biological replicates. Oligonucleotides used for qPCR assays are given in Supplementary Table 5.

References:

1. Kulshreshtha R *et al* (2007) A microRNA signature of hypoxia. *Mol Cell Biol* **27:** 1859-1867
2. Hua Z *et al* (2006) MiRNA-directed regulation of VEGF and other angiogenic factors under hypoxia. *PLoS One* **1:** e116
3. Hebert C, Norris K, Scheper MA, Nikitakis N, Sauk JJ (2007) High mobility group A2 is a target for miRNA-98 in head and neck squamous cell carcinoma. *Mol Cancer* **6:** 5
4. Donker RB, Mouillet JF, Nelson DM, Sadovsky Y (2007) The expression of Argonaute2 and related microRNA biogenesis proteins in normal and hypoxic trophoblasts. *Mol Hum Reprod* **13:** 273-279
5. Guimbellot JS, Erickson SW, Mehta T, Wen H, Page GP, Sorscher EJ, Hong JS (2009) Correlation of microRNA levels during hypoxia with predicted target mRNAs through genome-wide microarray analysis. *BMC Med Genomics* **2:** 15
6. Langmead B, Trapnell C, Pop M, Salzberg SL (2009) Ultrafast and memory-efficient alignment of short DNA sequences to the human genome. *Genome Biol* **10:** R25
7. Trapnell C, Williams BA, Pertea G, Mortazavi A, Kwan G, van Baren MJ, Salzberg SL, Wold BJ, Pachter L (2010) Transcript assembly and quantification by RNA-Seq reveals unannotated transcripts and isoform switching during cell differentiation. *Nat Biotechnol* **28:** 511-515
8. Kong L, Zhang Y, Ye ZQ, Liu XQ, Zhao SQ, Wei L, Gao G (2007) CPC: assess the protein-coding potential of transcripts using sequence features and support vector machine. *Nucleic Acids Res* **35:** W345-349
9. Schodel J, Oikonomopoulos S, Ragoussis J, Pugh CW, Ratcliffe PJ, Mole DR (2011) High-resolution genome-wide mapping of HIF-binding sites by ChIP-seq. *Blood* **117:** e207-217
