# Supplementary material for: Extensive regulation of the non-coding transcriptome by hypoxia: role of HIF in releasing paused RNApol2
Source: EMBO Rep. 2013 Dec 22;15(1):70–6. doi: 10.1002/embr.201337642 (PMC3983684; doi:10.1002/embr.201337642)
Supplement: Supplementary file 18 [file embr0015-0070-sd18.pdf]

EMBO
*reports*

Manuscript EMBOR-2013-37642

# Extensive regulation of the non-coding transcriptome by hypoxia: role of HIF in releasing paused RNApol2

Hani Choudhry, Johannes Schodel, Spyros Oikonomopoulos, Carme Camps, Steffen Grampp, Adrian L Harris, Peter J Ratcliffe, Jiannis Ragoussis and David R Mole

*Corresponding authors: David R Mole and Peter J Ratcliffe, University of Oxford, Jiannis Ragoussis, McGill University, Montreal*

**Review timeline:**

| | |
|---|---|
| Submission date: | 17 June 2013 |
| Editorial Decision: | 10 July 2013 |
| Revision received: | 30 September 2013 |
| Correspondence: | 9 October 2013 |
| Correspondence: | 18 October 2013 |
| Accepted: | 22 October 2013 |

## Transaction Report:

(Note: With the exception of the correction of typographical or spelling errors that could be a source of ambiguity, letters and reports are not edited. The original formatting of letters and referee reports may not be reflected in this compilation.)

Editor: Barbara Pauly

1st Editorial Decision 10 July 2013

Thank you very much for the submission of your research manuscript to our editorial office. We have now received the full set of reviews on your manuscript.

As the detailed reports are pasted below I will only repeat the main points here. You will see that the reviewers appreciate the interest of your findings and are, in principle, supportive of publication of your study in our journal. However, they also point out aspects of your study that would need to be further strengthened before publication. Both referee 1 and 3 point out several instances in which additional clarifications and discussions are needed, for example with regard to apparent inconsistencies between the miRNA data in the current study and in previous reports. Referee 1 also feels that additional insights into the biological significance of the hypoxia-induced upregulation of MALAT1 and NEAT1 should be provided. In addition, this reviewer also recommends testing whether there is a defect in hypoxia-induced polymerase release in cells lacking HIF proteins.

Given these positive evaluations, the reviewers constructive comments and the potential interest of the study, I would like to give you the opportunity to revise your manuscript, with the understanding

© European Molecular Biology Organization 

that the main concerns of the reviewers should be addressed. Acceptance of the manuscript will depend on a positive outcome of a second round of review and I should also remind you that it is EMBO reports policy to allow a single round of revision only and that therefore, acceptance or rejection of the manuscript will depend on the completeness of your responses included in the next, final version of the manuscript.

Revised manuscripts should be submitted within three months of a request for revision; they will otherwise be treated as new submissions. If you feel that this period is insufficient for a successful submission of your revised manuscript I can potentially extend this period slightly. Also, the length of the revised manuscript should not exceed roughly 29,000 characters (including spaces and references). If you feel that the additional data requested by the reviewers would make the manuscript too long you may consider including some peripheral data in the form of Supplementary information. However, Materials and Methods essential for the repetition of the main experiments should not be displayed as supplementary information only.

As part of the EMBO publication's Transparent Editorial Process, EMBO reports publishes online a Review Process File to accompany accepted manuscripts. This File will be published in conjunction with your paper and will include the referee reports, your point-by-point response and all pertinent correspondence relating to the manuscript.

You are able to opt out of this by letting the editorial office know (emboreports@embo.org). If you do opt out, the Review Process File link will point to the following statement: "No Review Process File is available with this article, as the authors have chosen not to make the review process public in this case."

We also welcome the submission of cover suggestions or motifs that might be used by our Graphics Illustrator in designing a cover.

I look forward to seeing a revised form of your manuscript when it is ready. Should you in the meantime have any questions, please do not hesitate to contact me.

REFEREE REPORTS:

Referee #1:

General Comments:

In this manuscript, Choudhry et al. have used integrated genomic (RNA-seq and ChIP-seq) and bioinformatic analysis to investigate the impact of hypoxia and the HIF transcription factor in the regulation of the coding and non-coding transcriptional response in a breast cancer cell line (MCF-7). The main conclusions are that all classes of RNA including both coding and non-coding RNA are regulated by hypoxia and that HIF plays a major role in this. Furthermore, the effects of HIF are hypothesized to be mediated through releasing pre-bound RNApol2. The regulation of the transcriptional response to hypoxia is an interesting and important area of research and the current study is of interest. However, the role of HIF in the regulation of the coding transcriptome has been previously characterized as has the impact of hypoxia and HIF in regulating non-coding RNAs such as miRNAs. Therefore, while the current study brings a strong -omic analysis of hypoxic signaling in regulating the non-coding transcriptome, the novelty in terms of biological insight provided is more limited.

Specific Points:

1) More clarity and a more convincing case needs to be made for the consistency (or not) of the current miRNA data with previously published work. For example, the authors refer to Ho et al. to state that mir-184 (found as the most up-regulated in the author's dataset) is consistent with previous work whereas in the Ho paper, mir-184 was in fact marginally down-regulated in hypoxia. In other words, how consistant are the pan-genomic analyses of the hypoxic response between studies and models, particularily in the context of miRNAs where a number of genomic studies have been published. Furthermore, the cell-type specificity of the most highly regulated miRNAs should be

tested by measuring levels of these transcripts in other cell types exposed to hypoxia.

2) The data in relation to MALAT1 and NEAT1 is overinterpreted without clear biological evidence. For example: "..the relative increase in expression of MALAT1 and NEAT1 in particular, likely reflect a previously unknown aspect of hypoxia biology in cancer". No evidence is provided for this. It is important to show some biologic effect of the hypoxia-induced MALAT1 or NEAT1 in a functional assay to substantiate in some part this claim.

3) In figure 2A, the number of binding sites in the vacinities of miRNAs appears to be very low relative to pcRNA and lncRNA. However the median fold regulation outlined in Figure 1 is similar for miRNA and pcRNA. How do the authors account for this apparent discrepancy?

4) Did the analysis carried out in Figure 3 hold true for the to upregulated miRNAs. Is there evidence that the top upregulated miRNAs are HIF 1 or HIF-2 dependent (or independent)?

5) Can the authors provide direct evidence that HIF plays a role in releasing paused RNApol2 by demonstrating in HIF-KO cells that paused RNApol2 release is reversed?

Referee #2:

The manuscript by Choudhry et al makes a strong case for widespread regulation of ncRNAs in the response to hypoxia. In particular the evidence for regulation of lncRNA is compelling and novel. The authors furthermore provide good evidence for up-regulation of many HIF responsive genes by release of promoter-proximal pausing by RNA pol II. Overall the conclusions are interesting and well supported by the data.
Minor points:
1. The statement "Actively transcribing RNApol2 recruits histone methyl transferases that trimethylated H3K4." (p. 10-11) should be referenced. The "spreading" of K4me3 into the body of genes induced by hypoxia was new to me. The authors imply that this phenomenon has been previously documented but I did not find any reference to it.

2. The use of the term "pcRNA", which I had not seen previously, rather than "mRNA" seemed unnecessary and obscure.

3. The nomenclature used in Supp Fig. 4 is confusing as the legend refers to sense and antisense while the figure itself refers to plus and minus.

Referee #3:

Comments on manuscript EMBOR-2013-37642V1 by Choudhry et al.
Extensive regulation of the non-coding transcriptome by hypoxia: role of HIF in releasing paused RNApol2

In this manuscript, Choudhry et al. report on the hypoxic regulation of ncRNA transcripts in a single cancer cell line (MCF-7). Their analyses revealed differential hypoxic regulation of sub-classes of ncRNA, identified several previously non-recognized lncRNAs, demonstrated the role of HIF-1/2 in transcriptional upregulation (but not downregulation) of lncRNAs as well as miRs, and, interestingly, showed that RNApol2 binding was increased under hypoxic conditions downstream of the TSS, but not at the TSS itself, of HIF regulated genes.

Comments:
1. p. 5) How was the threshold (number of reads) defined to exclude low abundance transcripts? Fig. 1C) In contrast to the description provided in the figure legend, there is no dotted line in this figure. According to supplementary methods, the vertical line indicates the threshold of low abundance transcripts. Why has it been chosen log10 of 1 for some but log10 of 2 for other classes of non-coding transcripts?

2. p. 6) Many more miRNAs than only miR-184 and miR-210 (e.g. miR-24, miR-29c, miR-103, miR-107, miR-127, miR-155, Let7) have been reported to be directly regulated by HIF. A more systematic comparison between the authors' findings and published data could be included.
3. p. 12) While the authors use plural form throughout this paragraph, Figs. 4D and 4E actually show only one single gene for each of the two classes described. Terms like "infrequent" should hence be quantified. The relevance of the presence or absence of TSS occupancy by RNApol2 with regard to the known regulation and function of these genes (aldolase A and adrenomedullin?) might be discussed.
4. Previous studies revealed hypoxic regulation of ncRNAs (e.g. BBA 2010, 1803, 443; Nucleic Acids Res. 2012, 40, 1928; Mol. Cell 2013, 49, 1083) and might be discussed in light of the authors' findings.

Minor points:
1. Fig. 3A) why is an approx. 10 kb region shown for NEAT1, which is actually much shorter as indicated by the horizontal line, whereas the region shown for MALAT1 corresponds to its gene length?
2. Fig. 4; Suppl. Figs. 7 and 8) define "FPKM".
3. p. 10) typo: ...HIF targets genes...
4. p. 10) "downregulated by HIF-1/2alpha" - specify that knock down of HIF-1/2alpha is actually meant.
5. p. 18) provide antibody identifiers.
6. Suppl. Fig. 2) disclose the genes analysed.
7. Suppl. Fig. 4C) HIF1A not HIF1a.
8. Suppl. Table 4) aSPAG4 is mentioned twice and the fwd and rev primers listed in the second line are identical.

## 1st Revision - authors' response — 30 September 2013

Referee #1:

General Comments:

In this manuscript, Choudhry et al. have used integrated genomic (RNA-seq and ChIP-seq) and bioinformatic analysis to investigate the impact of hypoxia and the HIF transcription factor in the regulation of the coding and non-coding transcriptional response in a breast cancer cell line (MCF-7). The main conclusions are that all classes of RNA including both coding and non-coding RNA are regulated by hypoxia and that HIF plays a major role in this. Furthermore, the effects of HIF are hypothesized to be mediated through releasing pre-bound RNApol2. The regulation of the transcriptional response to hypoxia is an interesting and important area of research and the current study is of interest. However, the role of HIF in the regulation of the coding transcriptome has been previously characterized as has the impact of hypoxia and HIF in regulating non-coding RNAs such as miRNAs. Therefore, while the current study brings a strong -omic analysis of hypoxic signaling in regulating the non-coding transcriptome, the novelty in terms of biological insight provided is more limited.

Specific Points:

1) More clarity and a more convincing case needs to be made for the consistency (or not) of the current miRNA data with previously published work. For example, the authors refer to Ho et al. to state that mir-184 (found as the most up-regulated in the author's dataset) is consistent with previous work whereas in the Ho paper, mir-184 was in fact marginally down-regulated in hypoxia. In other words, how consistant are the pan-genomic analyses of the hypoxic response between studies and models, particularily in the context of miRNAs

© European Molecular Biology Organization 

where a number of genomic studies have been published. Furthermore, the cell-type specificity of the most highly regulated miRNAs should be tested by measuring levels of these transcripts in other cell types exposed to hypoxia.

We thank the reviewer for pointing out that it is only the precursor mir-184 that is upregulated by hypoxia in the Ho paper and that in fact the mature mir-184 is marginally (although not significantly) downregulated after 24 hours hypoxia, when compared to normoxic levels. Indeed mir-210 was the only significantly upregulated mature miRNA in this study in HUVEC cells. We have revised the manuscript accordingly.

As suggested, we have now undertaken a more systematic comparison of our current miRNA data with previously published work. Specifically, we have related our findings to 5 previously published pan-genomic studies of hypoxia-regulated miRNAs and include this analysis as a new Supplementary Table 2. These previous papers have used assorted cell types, hypoxic conditions and profiling platforms and have exhibited a high degree of heterogeneity between studies. Indeed, in a systematic review of the literature (McCormick et al 2010) only 23/103 (22%) were regulated in more than one study with 9 of these (9%) regulated in opposing rather than congruent directions. In the light of this heterogeneity, our data shows similar levels of overlap with each of these previously published studies as was observed between the different studies.

In addition, we have now examined a selection of the most upregulated miRNAs from our analysis across a panel of breast cancer cell lines (MDA-MB-231, T47D, SKBR3, BT474 and MCF-7). Specifically, we have examined those miRNAs (mir-184, mir-1, mir-612, mir-100 and mir-210) for which validated TaqMan probes were available. All miRNAs tested were upregulated in MCF-7 cells by qPCR analysis. Each miRNA was also upregulated in between 2 and 4 out of the 4 additional cell lines. This data has been included in the revised manuscript as a new Supplemental Figure 3.

2) The data in relation to MALAT1 and NEAT1 is overinterpreted without clear biological evidence. For example: "..the relative increase in expression of MALAT1 and NEAT1 in particular, likely reflect a previously unknown aspect of hypoxia biology in cancer". No evidence is provided for this. It is important to show some biologic effect of the hypoxia-induced MALAT1 or NEAT1 in a functional assay to substantiate in some part this claim.

We agree with the reviewer that taken in isolation the current study does not provide direct evidence of a causal association between MALAT1 or NEAT1 and tumourigenesis. We have therefore amended our statement to avoid potential over-interpretion of the data. We do however provide reference to a growing body of published literature indicating a role for MALAT1 in tumourigenesis and tumour metastasis (e.g. Schmidt LH et al - 2011). Furthermore, we provide new data (Supplemental Figure 4) illustrating upregulation of both NEAT1 and MALAT1 in a panel of additional breast cancer cell lines. An in depth analysis of the biological effects of this hypoxia-induced NEAT1 and MALAT1 in breast cancer forms the basis of ongoing work, which we feel is beyond the scope of the current report encompassing our pan-genomic findings.

3) In figure 2A, the number of binding sites in the vacinities of miRNAs appears to be very low relative to pcRNA and lncRNA. However the median fold regulation outlined in Figure 1 is similar for miRNA and pcRNA. How do the authors account for this apparent discrepancy?

This likely reflects an artifact of the method by which the genomic location of a HIF-binding site is linked to a gene. In the absence of pan-genomic assessments of 3-dimensional chromatin conformation to identify looping between enhancers and promoters, the current standard method of associating enhancers with promoters for subsequent bioinformatic

© European Molecular Biology Organization

analysis is to use the closest expressed transcript to the transcription factor-binding site in question. For most classes of transcript this provides a good first approximation. However, the genomic locations of miRNAs are frequently embedded within a host gene, the promoter of which may be closer to the HIF-binding site than the miRNA itself. We have repeated the analysis looking for all transcripts within a fixed distance of a HIF-binding site. Whilst this does identify larger numbers of upregulated miRNAs that are associated with HIF-binding sites it also links other neighbouring non-regulated genes to these sites, diluting the statistical power of the GSEA analysis. Therefore in the interests of simplicity we have adhered to the same conventional method for all classes of gene. But have added a brief comment to the revised manuscript in explanation?

4) Did the analysis carried out in Figure 3 hold true for the top upregulated miRNAs. Is there evidence that the top upregulated miRNAs are HIF 1 or HIF-2 dependent (or independent)?

The RNA-seq analysis of the effects of HIF suppression were performed using polyA-selected RNA and so miRNAs were not detected. We are therefore unable to comment upon the effects of HIF siRNA on miRNAs and we have added a comment to this effect in the revision.

5) Can the authors provide direct evidence that HIF plays a role in releasing paused RNApol2 by demonstrating in HIF-KO cells that paused RNApol2 release is reversed?

This is an important question and we have now included new data to show that HIF plays a role in releasing RNApol2 in the main manuscript. We have suppressed the HIF pathway using combined HIF-1$\alpha$ and HIF-2$\alpha$ siRNA (method previously described in the manuscript) and controlled this with a non-targeting siRNA. Using hypoxic induction at the ALDOA and NDRG1 gene loci as examples of the release of paused RNApol2 we then determined levels of RNApol2 travelling across the gene body using RNApol2 ChIP-qPCR. As seen in the ChIP-seq analyses, hypoxia increased the levels of travelling RNApol2 within the body of the gene. Importantly, this induction was greatly reduced following the combined suppression of HIF-1$\alpha$ and HIF-2$\alpha$ indicating that the release of RNApol2 was dependent on HIF. Suppression of HIF had no effect on normoxic levels of RNApol2 travelling across the gene body. Similar results were observed at several positions within the gene body. We have added these new data as additional panels in Figure 4 of the main manuscript.

Referee #2:

The manuscript by Choudhry et al makes a strong case for widespread regulation of ncRNAs in the response to hypoxia. In particular the evidence for regulation of lncRNA is compelling and novel. The authors furthermore provide good evidence for up-regulation of many HIF responsive genes by release of promoter-proximal pausing by RNA pol II. Overall the conclusions are interesting and well supported by the data.

Minor points:

1. The statement "Actively transcribing RNApol2 recruits histone methyl transferases that trimethylated H3K4." (p. 10-11) should be referenced. The "spreading" of K4me3 into the body of genes induced by hypoxia was new to me. The authors imply that this phenomenon has been previously documented but I did not find any reference to it.

This has now been referenced. We have also updated the manuscript in the light of a new publication indicating that p300 (to which HIF binds) may also direct histone methyltransferase activity to active promoters.

© European Molecular Biology Organization 

2. The use of the term "pcRNA", which I had not seen previously, rather than "mRNA" seemed unnecessary and obscure.

We have amended the notation from pcRNA to mRNA

3. The nomenclature used in Supp Fig. 4 is confusing as the legend refers to sense and antisense while the figure itself refers to plus and minus.

The plus and minus refers to the reference genome (i.e whether the gene is transcribed from left-to-right or right-to-left), whilst the term sense and antisense refers to the annotated transcript at that locus (i.e whether the RNAs are transcribed from the same strand or opposing strands) and depends upon the DNA strand from which the annotated RNA is transcribed. Since each of the four examples shown is transcribed from the plus strand (i.e. transcribed from left-to-right), this distinction is not obvious and we apologize for the confusion. For consistency, we have amended the figure to read “sense” and “antisense” to make this clearer.

Referee #3:

Comments on manuscript EMBOR-2013-37642V1 by Choudhry et al.
Extensive regulation of the non-coding transcriptome by hypoxia: role of HIF in releasing paused RNApol2

In this manuscript, Choudhry et al. report on the hypoxic regulation of ncRNA transcripts in a single cancer cell line (MCF-7). Their analyses revealed differential hypoxic regulation of sub-classes of ncRNA, identified several previously non-recognized lncRNAs, demonstrated the role of HIF-1/2 in transcriptional upregulation (but not downregulation) of lncRNAs as well as miRs, and, interestingly, showed that RNApol2 binding was increased under hypoxic conditions downstream of the TSS, but not at the TSS itself, of HIF regulated genes.

Comments:
1. p. 5) How was the threshold (number of reads) defined to exclude low abundance transcripts? Fig. 1C) In contrast to the description provided in the figure legend, there is no dotted line in this figure. According to supplementary methods, the vertical line indicates the threshold of low abundance transcripts. Why has it been chosen log10 of 1 for some but log10 of 2 for other classes of non-coding transcripts?

Plotting the fold regulation against the number of reads for each transcript revealed that below 10 reads the results for fold-regulation were more widely scattered (Figure 1c) and so these transcripts were excluded from further analysis. Since protein coding RNAs and lncRNAs are longer transcripts than the other classes we used a higher cut off for these two classes (i.e log10 of 2 = 100 reads, rather than log10 of 1 = 10 reads). Whilst the use of these cutoffs reduces the noise and increases the certainty of the result, the overall changes observed were robust to the precise threshold used. We have added a brief note in figure legend

2. p. 6) Many more miRNAs than only miR-184 and miR-210 (e.g. miR-24, miR-29c, miR-103, miR-107, miR-127, miR-155, Let7) have been reported to be directly regulated by HIF. A more systematic comparison between the authors' findings and published data could be included.

The reviewer is correct in pointing out that many miRNAs have been identified as participating in the hypoxic response. Indeed there have been a number of microarray-based pan-genomic analyses of the miRNA response and we had therefore chosen to focus our analysis on the lncRNA response. We have now undertaken a systematic comparison of our data with the findings from 5 published microarray based studies and

have included this in the supplemental data (Supplemental Table 2). Despite significant heterogeneity between the published reports, the degree of overlap between our analysis and microarray-based approaches is comparable to that between these previous studies. Interestingly, among the examples listed by the reviewer above we see strong regulation of miR-24, although we have identified HIF binding at the 24-2 locus rather than the postulated 24-1 locus that was suggested by exogenous reporter assays.

3. p. 12) While the authors use plural form throughout this paragraph, Figs. 4D and 4E actually show only one single gene for each of the two classes described. Terms like "infrequent" should hence be quantified. The relevance of the presence or absence of TSS occupancy by RNApol2 with regard to the known regulation and function of these genes (aldolase A and adrenomedullin?) might be discussed.

Since we see multiple examples of each type of behaviour, the use of the plural term is correct. However, in the interests of brevity and to avoid duplication, we have only illustrated one example of each extreme of behavior.

Whilst we have illustrated the two extremes of behavior, the *de novo* recruitment of RNApol2 to the promoters of hypoxically induced genes follows a continuum, with some genes showing intermediate behavior (i.e. a small amount of RNApol2 at the promoter in normoxia, which increases in hypoxia). Overall, the *de novo* recruitment of RNApol2 is an infrequent occurrence, since the average change in RNApol2 at the promoters of hypoxically induced genes changes very little (Figure 4A). However, it is difficult to be precise about the exact numbers, since the classification of genes into one of two categories based on a continuous variable would depend greatly on the threshold used to define these categories. Therefore, rather than providing a potentially misleading numerical quantitation, we have deliberately used more qualitative terms.

The question of whether genes from different biological pathways are regulated in different ways is an intriguing one. However, when we looked to see whether there was any correlation between RNApol2 pause-release or *de novo* recruitment and biological function (using Ingenuity Pathways Analysis), we were not able to see any significant association. We have added a comment to this effect in the manuscript.

4. Previous studies revealed hypoxic regulation of ncRNAs (e.g. BBA 2010, 1803, 443; Nucleic Acids Res. 2012, 40, 1928; Mol. Cell 2013, 49, 1083) and might be discussed in light of the authors' findings.

We thank the reviewer for highlighting these examples and references. In particular, H19 was upregulated by hypoxia in our analysis, but without a HIF binding site close to the gene. MALAT1 and NEAT1, both found to be upregulated by Wollenick (NAR 2012, 40, 1928), were the two most upregulated HIF binding lncRNAs in our dataset. We have included discussion of our findings in relation to these previous reports in the manuscript. LET was not expressed in our dataset and has not been discussed.

Minor points:

1. Fig. 3A) why is an approx. 10 kb region shown for NEAT1, which is actually much shorter as indicated by the horizontal line, whereas the region shown for MALAT1 corresponds to its gene length?

A longer region than the annotated transcript is included for NEAT1 to show the entirety of an unannotated species identified in the ribosome depleted RNA-seq, which by reference to the polyA+ RNA-seq signal is predominantly non-polyadenylated.

2. Fig. 4; Suppl. Figs. 7 and 8) define "FPKM".

FPKM refers to the standard notation for “fragments per kilobase per million reads” (i.e. normalized to genomic interval and to read depth between samples) and we have now clarified this in the figure legend.

3. p. 10) typo: ...HIF targets genes...

Thank you. We have corrected this.

4. p. 10) "downregulated by HIF-1/2alpha" - specify that knock down of HIF-1/2alpha is actually meant.

Thank you. We have amended this to read “downregulated by HIF-1/2alpha siRNA” etc.

5. p. 18) provide antibody identifiers.

Suppliers and catalogue numbers have now been included in the text.

6. Suppl. Fig. 2) disclose the genes analysed.

This has now been included in the figure legend.

7. Suppl. Fig. 4C) HIF1A not HIF1a.

This has been amended.

8. Suppl. Table 4) aSPAG4 is mentioned twice and the fwd and rev primers listed in the second line are identical.

This has been corrected.

Correspondence - Editor 9 October 2013

---

Many thanks for submitting your revised manuscript to EMBO reports. I am happy to let you know that the two referees who were asked to assess it now support publication in our journal.

Before we proceed with the official acceptance I would, however, kindly ask you to clarify two additional points:

1. For experiments in which you have calculated error bars (I think this applies to figure 1D and 4D and E), please indicate in the respective figure legend whether you have performed these experiments in duplicated or triplicate. Please also note that error bars should only be calculated if the experiments have done at least three times (biological, not technical replicates).

2. For the RNA-seq and ChIP-seq data, please indicate exactly where they are available (currently it only says 'at NCBI GEO (XXXXXXX)"

3. Please send us a short, two-sentence summary of your work for our website.

You can send the modified text/figures as email attachments as this seems to be easiest.

Correspondence – Authors reply 18 October 2013

Many thanks for your consideration of our manuscript and we are delighted that you have chosen to publish our findings. I apologise for the delay in replying, but we have had to deposit our raw data at EMBL-EBI Array Express as the NCBI site has been closed to new depositions.

With regard to your specific points:

1. Figures 4D and 4E were performed as technical triplicates on two biological replicates and I have therefore removed the error bars (New Figure 4 appended) and specified two biological duplicates in the figure legend as per your instruction. Figure 1D is a box-and-whisker plot showing the interquartile and full-range of 100's to 1000's genes within each RNA sub-type. All other figures, including supplemental figures were performed as biological triplicates and this is specified in the respective figure legends.

2. The RNA-seq and ChIP-seq datasets have now been deposited at EMBL-EBI Array Express (accession numbers E-MTAB-1994, E-MTAB-1995) and the manuscript has been amended to include this.

3. A short two-sentence summary is included below:
"Integrated genomic analysis of the hypoxia response using massively parallel sequencing identifies profound regulation of the non-coding as well as the coding transcriptome. The release of pre-bound promoter-paused RNApol2 by hypoxia-inducible factor (HIF) is a major effector of this transcriptional response."
I am attaching the modified manuscript to this email and will send the revised figure 4 as a separate email as it is over 10 Mb in size. Please let me know if there is anything else that you require.

2nd Editorial Decision 22 October 2013

I am very pleased to accept your manuscript for publication in the next available issue of EMBO reports. Thank you for your contribution to our journal.

As part of the EMBO publication's Transparent Editorial Process, EMBO reports publishes online a Review Process File to accompany accepted manuscripts. As you are aware, this File will be published in conjunction with your paper and will include the referee reports, your point-by-point response and all pertinent correspondence relating to the manuscript.

If you do NOT want this File to be published, please inform the editorial office within 2 days, if you have not done so already, otherwise the File will be published by default [contact: emboreports@embo.org]. If you do opt out, the Review Process File link will point to the following statement: "No Review Process File is available with this article, as the authors have chosen not to make the review process public in this case."

Thank you again for your contribution to EMBO reports and congratulations on a successful publication. Please consider us again in the future for your most exciting work.
